# HbA_1c_ levels in non-diabetic older adults – No J-shaped associations with primary cardiovascular events, cardiovascular and all-cause mortality after adjustment for confounders in a meta-analysis of individual participant data from six cohort studies

**DOI:** 10.1186/s12916-016-0570-1

**Published:** 2016-02-11

**Authors:** Ben Schöttker, W. Rathmann, C. Herder, B. Thorand, T. Wilsgaard, I. Njølstad, G. Siganos, E. B. Mathiesen, K. U. Saum, A. Peasey, E. Feskens, P. Boffetta, A. Trichopoulou, K. Kuulasmaa, F. Kee, H. Brenner, on behalf of the CHANCES group

**Affiliations:** Division of Clinical Epidemiology and Aging Research, German Cancer Research Center, Im Neuenheimer Feld 581, 69120 Heidelberg, Germany; Network Aging Research, University of Heidelberg, Bergheimer Straße 20, 69115 Heidelberg, Germany; Institute for Biometrics and Epidemiology, German Diabetes Center, Leibniz Center for Diabetes Research at Heinrich Heine University Düsseldorf, Auf`m Hennekamp 65, 40225 Düsseldorf, Germany; Institute for Clinical Diabetology, German Diabetes Center, Leibniz Center for Diabetes Research at Heinrich Heine University Düsseldorf, Auf`m Hennekamp 65, 40225 Düsseldorf, Germany; German Center for Diabetes Research (DZD), Ingolstädter Landstraße 1, 85764 München-Neuherberg, Germany; Institute of Epidemiology II, Helmholtz Zentrum München, German Research Center for Environmental Health, Postfach 1129, Neuherberg, Germany; Epidemiology of Chronic Diseases Research Group, Department of Community Medicine, UiT The Arctic University of Norway, 9037 Tromsø, Norway; Brain and Circulation Research Group, Department of Clinical Medicine, UiT The Arctic University of Norway, 9037 Tromsø, Norway; Department of Epidemiology and Public Health, University College London, 1–19 Torrington Place, London, WC1E 6BT UK; Division of Human Nutrition, Wageningen University, PO Box 8129, 6700 EV Wageningen, The Netherlands; Institute for Translational Epidemiology and The Tisch Cancer Institute, Icahn School of Medicine at Mount Sinai, New York, NY USA; Hellenic Health Foundation, Kaisareias 13 and Alexandroupoleos, Athens, 11527 Greece; National Institute for Health and Welfare (THL), PO Box 30, FI-00271 Helsinki, Finland; UKCRC Centre of Excellence for Public Health, Queen’s University Belfast, Belfast, Northern Ireland

**Keywords:** Glycated hemoglobin, Cardiovascular disease, Myocardial infarction, Stroke, Mortality, Cohort study, Meta-analysis

## Abstract

**Background:**

To determine the shape of the associations of HbA_1c_ with mortality and cardiovascular outcomes in non-diabetic individuals and explore potential explanations.

**Methods:**

The associations of HbA_1c_ with all-cause mortality, cardiovascular mortality and primary cardiovascular events (myocardial infarction or stroke) were assessed in non-diabetic subjects ≥50 years from six population-based cohort studies from Europe and the USA and meta-analyzed. Very low, low, intermediate and increased HbA_1c_ were defined as <5.0, 5.0 to <5.5, 5.5 to <6.0 and 6.0 to <6.5 % (equals <31, 31 to <37, 37 to <42 and 42 to <48 mmol/mol), respectively, and low HbA_1c_ was used as reference in Cox proportional hazards models.

**Results:**

Overall, 6,769 of 28,681 study participants died during a mean follow-up of 10.7 years, of whom 2,648 died of cardiovascular disease. Furthermore, 2,493 experienced a primary cardiovascular event. A linear association with primary cardiovascular events was observed. Adjustment for cardiovascular risk factors explained about 50 % of the excess risk and attenuated hazard ratios (95 % confidence interval) for increased HbA_1c_ to 1.14 (1.03–1.27), 1.17 (1.00–1.37) and 1.19 (1.04–1.37) for all-cause mortality, cardiovascular mortality and cardiovascular events, respectively. The six cohorts yielded inconsistent results for the association of very low HbA_1c_ levels with the mortality outcomes and the pooled effect estimates were not statistically significant. In one cohort with a pronounced J-shaped association of HbA_1c_ levels with all-cause and cardiovascular mortality (NHANES), the following confounders of the association of very low HbA_1c_ levels with mortality outcomes were identified: race/ethnicity; alcohol consumption; BMI; as well as biomarkers of iron deficiency anemia and liver function. Associations for very low HbA_1c_ levels lost statistical significance in this cohort after adjusting for these confounders.

**Conclusions:**

A linear association of HbA_1c_ levels with primary cardiovascular events was observed. For cardiovascular and all-cause mortality, the observed small effect sizes at both the lower and upper end of HbA_1c_ distribution do not support the notion of a J-shaped association of HbA_1c_ levels because a certain degree of residual confounding needs to be considered in the interpretation of the results.

**Electronic supplementary material:**

The online version of this article (doi:10.1186/s12916-016-0570-1) contains supplementary material, which is available to authorized users.

## Background

Glycated hemoglobin (HbA_1c_) is a biomarker for impaired glucose metabolism and indicates the average blood glucose concentration over the previous 2–3 months [1]. Non-diabetic subjects with increased HbA_1c_, often termed “pre-diabetes”, do not only have a higher risk for the development of manifest diabetes mellitus but also for cardiovascular events and all-cause mortality [2–5]. However, it is unclear how much of the excess risk is contributed by the impaired glucose metabolism and how much by simultaneously increased cardiovascular risk factor levels [6, 7].

With regard to very low HbA_1c_ levels in subjects without diabetes mellitus, the results from population-based cohort studies are inconsistent. Whereas some authors did not observe an increased risk or observed a decreased risk [8–12], others reported an increased risk for cardiovascular outcomes or death among subjects with HbA_1c_ levels below 4.0 % (20 mmol/mol) [13], 4.8 % (29 mmol/mol) [14], 4.9 % (30 mmol/mol) [15] or 5.0 % (31 mmol/mol) [5, 16–18] compared to subjects from the adjacent higher HbA_1c_ interval, along with a stronger increase in risk in non-diabetic subjects with increased HbA_1c_. Some authors have called this a J-shaped or U-shaped relationship between HbA_1c_ and cardiovascular risk or mortality [5, 14, 16–18]. A statistically significant increased cardiovascular disease risk for non-diabetics with very low HbA_1c_ levels was for the first time observed in the largest study of this type to date, the individual participant data meta-analysis of 24 studies of the Emerging Risk Factors Collaboration (ERFC) [5]. So far, however, no study has aimed to assess potential explanations for an increased cardiovascular risk of non-diabetics with very low HbA_1c_ in a large consortium. Explanatory hypotheses, proposed in literature, include:

1. Underweight (as part of the frailty syndrome in older non-diabetics [19])
2. Inflammation (caused by frequent asymptomatic hypoglycemic episodes [20])
3. Anemia with or without iron deficiency (because of abnormalities of erythrocyte indices [21] and potentially correlated hemoglobin and HbA_1c_ values [22])
4. High alcohol consumption (inhibiting the gluconeogenesis in the liver [23] and shortening the red blood cell lifespan)
5. Liver disease (as a result of high alcohol consumption [17])
6. Chronic renal failure (reduced red blood cell lifespan and increased carbamylated hemoglobin affect the accuracy of HbA_1c_ measurements [24, 25])
7. Hematologic differences according to race/ethnicity (non-Hispanic black people have a special hematologic profile [26] and have more frequently very low HbA_1c_ levels than people of other race/ethnicity [27]).

The objective of this meta-analysis of individual participant data is to investigate whether there is a J-shaped association of HbA_1c_ levels with cardiovascular events, cardiovascular mortality and all-cause mortality in non-diabetic older adults and to explore potential explanations for a potentially increased risk at very low HbA_1c_ levels.

## Methods

### Study design and study population

This investigation is a meta-analysis of individual participant data of six population-based cohort studies: Tromsø (Norway); ELSA (UK); NHANES (USA); ESTHER (Germany); KORA (Germany); and SHIP (Germany). Details of each study’s acronym, recruitment procedure and data collection are given in Additional file 1. All variables were harmonized in the framework of the Consortium on Health and Ageing: Network of Cohorts in Europe and the United States (CHANCES; www.chancesfp7.eu).

### Ethics, consent and permissions

The included studies have been approved by local ethics committees (see Additional file 1). Written informed consent has been obtained from all participants included in the analyzed studies and the studies are being conducted in accordance with the Declaration of Helsinki.

### Inclusion and exclusion criteria

Study participants with missing HbA_1c_ measurements at baseline were excluded. To make cohorts more comparable, analyses were restricted to study participants aged 50 years and older. Furthermore, to restrict the sample to subjects without diabetes mellitus, all subjects with diagnosed diabetes mellitus, missing information about a diabetes diagnosis and with potential undiagnosed diabetes (defined by HbA_1c_ ≥6.5 % (≥48 mmol/mol) [28]) were excluded. The final number of included study participants from each cohort is shown in Table 1. The sample sizes differ for the outcomes because subjects lost to follow-up right after baseline were excluded. Subjects who died of unknown causes were only excluded for the outcome “cardiovascular mortality” (ICD-10 code R96–99 or missing) and subjects with a history of myocardial infarction (MI) or stroke at baseline were only excluded for the outcome “primary cardiovascular events”.

**Table 1:** Baseline characteristics of participants without diabetes mellitus and number of events during follow-up of included cohort studies

Baseline characteristic	Unit	ESTHER	SHIP	ELSA	Tromsø	KORA	NHANES
Baseline years for this analysis		2000–2002	1997–2001	2004–2005	1994–1995	1999–2001	1988–1994
Total sample size		7,982	1,777	5,262	6,045	1,850	5,778
Age	Years	61.8 (6.6)	63.4 (8.3)	65.8 (9.7)	61.6 (7.0)	61.7 (7.1)	67.9 (11.0)
Age ≥65 years	%	2,481 (31.1)	733 (41.3)	2,410 (45.8)	1,958 (32.4)	645 (34.9)	3,157 (54.6)
Male sex	%	3,476 (43.6)	905 (50.9)	2,334 (44.4)	2,550 (42.2)	929 (50.2)	2,774 (48.0)
Race/ethnicity							
Non-Hispanic white	%	N/A	N/A	N/A	N/A	N/A	3,428 (60.3)
Non-Hispanic black	%	N/A	N/A	N/A	N/A	N/A	1,088 (18.8)
Mexican-American	%	N/A	N/A	N/A	N/A	N/A	997 (17.3)
Other	%	N/A	N/A	N/A	N/A	N/A	211 (3.7)
School education							
≤9 years	%	5,743 (73.5)	975 (55.2)	968 (19.0)	3,228 (53.8)	322 (17.5)	2,238 (39.1)
10–12 years	%	1,671 (21.4)	443 (25.1)	3,137 (61.6)	1,773 (29.5)	1,156 (62.7)	2,147 (37.5)
≥13 years	%	398 (5.1)	348 (19.7)	992 (19.5)	1,005 (16.7)	367 (19.9)	1,343 (23.5)
BMI	kg/m^2^	27.3 (4.2)	28.2 (4.4)	27.5 (4.6)	25.9 (3.8)	28.1 (4.2)	26.8 (5.2)
BMI category							
Underweight	%	146 (1.8)	29 (1.6)	141 (2.8)	239 (4.0)	21 (1.1)	361 (6.3)
Optimal weight	%	2,249 (28.2)	389 (21.9)	1,356 (26.9)	2,360 (39.1)	389 (21.2)	1,835 (31.9)
Overweight	%	3,816 (47.9)	832 (46.9)	2,247 (44.6)	2,603 (43.2)	907 (49.3)	2,251 (39.2)
Obese	%	1.763 (22.1)	525 (29.6)	1,296 (25.7)	831 (13.8)	522 (28.4)	1,303 (22.7)
Smoking							
Never	%	3,974 (51.1)	942 (54.6)	2,390 (45.8)	1,949 (32.3)	969 (53.4)	2,583 (44.7)
Former	%	2,479 (31.9)	486 (28.2)	1,922 (36.8)	2,184 (36.2)	568 (31.3)	1,956 (33.9)
Current	%	1,319 (17.0)	298 (17.3)	905 (17.4)	1,907 (31.6)	279 (15.4)	1,239 (21.4)
Relative alcohol consumption							
Abstainer or low	%	3,851 (53.2)	848 (50.5)	2,537 (50.1)	2,394 (51.7)	1,005 (54.6)	3,410 (64.2)
Moderate	%	2,650 (36.6)	655 (39.0)	2,024 (39.9)	1,745 (37.7)	638 (34.6)	1,325 (24.9)
High	%	741 (10.2)	177 (10.5)	508 (10.0)	490 (10.6)	199 (10.8)	580 (10.9)
Vigorous physical activity	%	3,452 (43.4)	905 (51.5)	2,232 (42.4)	2,043 (34.2)	819 (44.5)	2,204 (38.2)
Total cholesterol	mmol/L	5.7 (1.3)	6.1 (1.2)	6.0 (1.2)	6.8 (1.3)	6.3 (1.1)	5.7 (1.1)
HDL cholesterol	mmol/L	1.4 (0.4)	1.5 (0.4)	1.5 (0.4)	1.6 (0.4)	1.5 (0.4)	1.4 (0.4)
Subclinical inflammation	%	2,786 (35.5)	492 (28.8)	1,827 (35.0)	1,079 (20.8)	520 (28.4)	2,187 (38.9)
Serum creatinine	nmol/L	79.8 (28.1)	87.1 (18.5)	N/A	67.6 (16.0)	76.5 (21.3)	101.6 (29.4)
Albuminuria	%	760 (9.6)	331 (21.0)	N/A	675 (12.8)	N/A	1,166 (22.0)
Hemoglobin	g/dL	N/A	13.6 (1.2)	14.3 (1.4)	14.1 (1.1)	14.4 (1.2)	13.9 (1.4)
Biomarkers of iron deficiency							
Ferritin	μg/L	N/A	N/A	N/A	N/A	N/A	157 (168)
Transferrin saturation	%	N/A	N/A	N/A	N/A	N/A	25.2 (10.6)
Erythrocyte protoporphyrin	μmol/L	N/A	N/A	N/A	N/A	N/A	0.95 (0.56)
Hypertension							
No hypertension	%	3,270 (41.0)	807 (45.5)	2,526 (48.0)	2,584 (42.8)	849 (46.1)	2,553 (44.2)
Known hypertension or systolic blood pressure ≥140 to <160 mmHg	%	3,915 (49.1)	593 (33.4)	2,313 (44.0)	2,044 (33.8)	778 (42.2)	2,545 (44.1)
Systolic blood pressure ≥160 mmHg	%	796 (10.0)	375 (21.1)	423 (8.0)	1,417 (23.4)	216 (11.7)	679 (11.8)
History of MI or stroke	%	511 (6.5)	140 (7.9)	398 (7.6)	438 (7.3)	97 (5.2)	738 (12.8)
Biomarkers of liver function							
GGT	U/L	N/A	N/A	N/A	N/A	N/A	31.6 (45.5)
AST	U/L	N/A	N/A	N/A	N/A	N/A	21.9 (11.8)
ALT	U/L	N/A	N/A	N/A	N/A	N/A	14.7 (10.6)
HbA_1c_	%	5.5 (0.4)	5.4 (0.5)	5.4 (0.3)	5.4 (0.4)	5.6 (0.3)	5.5 (0.5)
Very low (<5.0 % (<31 mmol/mol))	%	476 (6.0)	336 (18.9)	345 (6.6)	626 (10.4)	63 (3.4)	678 (11.7)
Low (5.0 to <5.5 % (31 to <37 mmol/mol))	%	2,747 (34.4)	546 (30.7)	2,344 (44.6)	2,719 (45.0)	603 (32.6)	1,994 (34.5)
Intermediate (5.5 to <6.0 % (37 to <42 mmol/mol))	%	3,726 (46.7)	583 (32.8)	2,201 (41.8)	2,323 (38.4)	937 (50.7)	2,272 (39.3)
Increased (6.0 to <6.5 % (42 to <48 mmol/mol))	%	1,033 (12.9)	312 (17.6)	372 (7.1)	377 (6.2)	247 (13.4)	834 (14.4)
Follow-up							
All-cause mortality							
Total sample size		7,981	1,777	5,253	6,045	1,850	5,775
Cases (%)		1,069 (13.4)	298 (16.8)	645 (12.3)	1,704 (28.2)	180 (9.7)	2,873 (49.8)
Mean FUP (SD)	Years	11.1 (2.1)	9.4 (2.1)	7.0 (1.3)	14.0 (3.8)	8.5 (1.3)	11.4 (4.9)
Cardiovascular mortality							
Total sample size		7,943	1,761	5,113	5,987	1,843	5,721
Cases (%)		263 (3.3)	90 (5.1)	174 (3.0)	707 (11.8)	69 (3.7)	1,345 (23.5)
Mean FUP (SD)	Years	11.1 (2.0)	9.4 (2.1)	7.0 (1.3)	14.1 (3.8)	8.6 (1.3)	11.4 (4.9)
Cardiovascular events							
Total sample size		7,233	1,637	4,462	5,602	1,556	N/A
Cases (%)		595 (8.2)	65 (4.0)	315 (7.1)	1,386 (24.7)	132 (8.5)	N/A
Mean FUP (SD)	Years	7.1 (2.3)	9.5 (2.0)	5.3 (1.4)	12.6 (4.8)	8.2 (1.7)	N/A

Unless indicated otherwise, the table shows proportions (%) for categorical and means (SD) for continuous variables. Numbers shown were drawn from the unimputed data set. Therefore, numbers do not always add up to the total because of missing values (see Additional file 1: Table S2 for number of missing values for each variable). ALT, alanine transferase; AST, aspartate transferase; BMI, body mass index; FUP, follow-up; GGT, gamma-glutamyl transferase; HbA_1c_, glycated hemoglobin; HDL, high-density lipoprotein; MI, myocardial infarction; N/A, not assessed; SD, standard deviation

### Outcome ascertainment

We assessed three outcomes: all-cause mortality; cardiovascular mortality; and primary cardiovascular events. The latter was defined by non-fatal MI, non-fatal stroke or cardiovascular death during follow-up, while subjects with a history of MI or stroke before baseline were excluded. Details about the assessment of the outcomes are outlined in the cohort descriptions (Additional file 1). In brief, all cohorts ascertained deaths by region- or country-wide registries. Data for incident non-fatal MI or stroke cases were available from all cohorts except NHANES. Diagnoses were based on medical records in Tromsø, ESTHER and KORA and on participant-reported physician diagnoses in ELSA and SHIP. If assessed, ICD-10 codes were used to ascertain cardiovascular mortality (I00–I99), MI (I21–I23) and stroke (I60–I69).

### Measurement of HbA_1c_

All cohorts measured HbA_1c_ with assays certified by the National Glycohemoglobin Standardization Program (NGSP), which are traceable to the assay of the Diabetes Control and Complications Trial (DCCT). Details about the assays are given in the cohort descriptions (Additional file 1).

### Covariate assessment

Socio-demographic, lifestyle, anthropometric and history of disease data were assessed by self-administered questionnaires or in interviews. In addition to self-reported information, some studies measured weight and height and validated the history of MI or stroke by consulting medical records or registries (Additional file 1: Table S1). If measured anthropometric data or validated diagnoses were available, they were used in the analysis and self-reported information was only used to fill missing information. With a modification of the underweight category, BMI categories of the World Health Organization (WHO) were applied to define underweight (<20 kg/m^2^), optimal BMI (20 to <25 kg/m^2^), overweight (25 to <30 kg/m^2^) and obesity (≥30 kg/m^2^). The underweight category was extended for our population of older adults because it has been previously shown that mortality is already increased at BMI <20 kg/m^2^ in individuals aged ≥65 years [29]. Total cholesterol, high-density lipoprotein (HDL) cholesterol, C-reactive protein (CRP), serum creatinine, urinary albumin and blood hemoglobin were measured for the total cohorts by routine methods in central laboratories cooperating with the study centers. Serum creatinine was not assessed in ELSA, blood hemoglobin was not measured in ESTHER and urinary albumin was not determined in ELSA and KORA. Subclinical inflammation was defined by CRP ≥3 mg/L [30] and albuminuria by urinary albumin ≥20 mg/L [31]. Biomarkers of liver function (alanine transferase (ALT), aspartate transferase (AST) and gamma-glutamyl transferase (GGT)) and iron deficiency (ferritin, transferrin saturation and erythrocyte protoporphyrin [32]) were only utilized from NHANES because this was the only study to assess all such indices. The analytical methods have been described elsewhere [32, 33]. Race/ethnicity was recorded in NHANES by four categories: non-Hispanic white; non-Hispanic black; Mexican-American; and other. The European studies included almost exclusively Caucasians and further differentiation of race/ethnicity in these cohorts was waived.

The different school-leaving qualifications of the countries were translated into the number of school years attended and three categories of education were devised (≤9 years, 10–12 years and ≥13 years). Reported average amounts of consumed wine, beer and spirits were converted into grams of pure ethanol per day and summed. Although the WHO reports comparable figures of consumed alcohol volumes per inhabitant in the European Union and United States [34], the calculated numbers from the cohorts diverged. In order to further standardize alcohol consumption, cohort and sex-specific percentiles (pct.) were calculated according to the average daily ethanol consumption and the following three categories of relative alcohol consumption were built: abstainer or low alcohol consumption (≤50th pct.); moderate alcohol consumption (>50th to <90th pct.); and high alcohol consumption (≥90th pct.). Vigorous physical activity was harmonized as a dichotomous variable (Yes or No) from questions regarding whether study participants perform physical activity that causes sweating (e.g. sports).

### Statistical analyses

Based on subject matter knowledge, HbA_1c_ levels were categorized in 0.5 % intervals and classified as “very low” (<5.0 % (31 mmol/mol)), “low” (5.0 to <5.5 % (31 to <37 mmol/mol)), “intermediate” (5.5 to <6.0 % (37 to <42 mmol/mol)) and “increased” (6.0 to <6.5 % (42 to <48 mmol/mol)). Individuals with low HbA_1c_ levels were used as the reference group in all analyses. Means or proportions with 95 % confidence intervals of baseline characteristics were calculated in each cohort stratified by HbA_1c_ category and pooled with a fixed effects model. In addition to these descriptive statistics, a multivariable logistic regression model was carried out with HbA_1c_ category as the dependent variable (with reference to the low HbA_1c_ group) and the baseline characteristics of the “full” model as independent variables. The “full” model comprised the variables of age, sex, race (for NHANES), BMI, education, smoking, physical activity, alcohol consumption, total cholesterol, HDL cholesterol, CRP, blood hemoglobin concentration, serum creatinine, albuminuria, hypertension, history of MI or stroke and biomarkers of liver function or iron deficiency. Variables were modelled continuously or in the categories shown in Table 2. Blood hemoglobin concentration, urinary albumin, serum creatinine and biomarkers of liver function or iron deficiency were not assessed in all cohorts (see Table 1) and were excluded from the “full” model for the respective cohorts.

**Table 2:** Baseline characteristics of subjects without diabetes mellitus by very low and low HbA_1c_ levels and multivariable adjusted odds ratios for associations of characteristics with very low HbA_1c_ levels. Pooled data from six cohort studies

Characteristic	Unit	Weighted mean or proportion (%) (95 % CI)		Pooled odds ratio (95 % CI)
		Very low HbA_1c_	Low HbA_1c_	
		(<5.0 %)	(5.0 to <5.5 %)	
		(<31 mmol/mol)	(31 to <37 mmol/mol) (reference group)	
Age	Years	61.1 (60.8–61.4)	62.2 (62.1–62.4)	**0.77 (0.72; 0.81) per 10 years**
Male sex	%	49.4 (47.4–51.3)	45.0 (44.1–46.0)	1.15 (0.93; 1.42)^a^
Race/ethnicity^b^				
Non-Hispanic white	%	63.3	70.0	Ref
Non-Hispanic black	%	20.2	11.8	**1.60 (1.21; 2.12)**
Mexican-American	%	13.7	15.1	0.88 (0.65; 1.17)
Other	%	2.8	3.0	0.88 (0.51; 1.53)
School education				
≤9 years	%	45.5 (43.4–47.6)	45.9 (44.9–46.9)	Ref
10–12 years	%	36.3 (34.3–38.2)	39.1 (38.1–40.0)	0.98 (0.91; 1.05)
≥13 years	%	21.4 (19.8–23.1)	19.0 (18.3–19.8)	1.00 (0.92; 1.09)
BMI category				
Underweight	%	4.5 (3.7–5.4)	3.9 (3.5–4.3)	1.13 (0.94; 1.35)
Optimal weight	%	36.2 (34.3–38.1)	34.6 (33.7–35.5)	Ref
Overweight	%	44.6 (42.7–46.6)	43.6 (42.7–44.6)	1.02 (0.93; 1.11)
Obese	%	16.0 (14.6–17.5)	19.2 (18.5–20.0)	**0.82 (0.73; 0.92)**
Smoking				
Never	%	46.4 (44.4–48.4)	45.8 (44.8–46.7)	Ref
Former	%	35.3 (33.5–37.2)	34.8 (33.9–35.7)	0.94 (0.85; 1.05)
Current	%	19.6 (18.0–21.3)	20.0 (19.5–21.1)	**0.70 (0.61; 0.81)**
Relative alcohol consumption				
Abstainer or low	%	48.0 (45.9–50.0)	50.9 (49.9–51.9)	Ref
Moderate	%	36.8 (34.8–38.8)	37.4 (36.4–38.3)	0.93 (0.87; 1.01)
High	%	15.7 (14.3–17.3)	12.0 (11.4–12.7)	**1.21 (1.10; 1.33)**
Vigorous physical activity	%	43.6 (41.6–45.5)	42.3 (41.4–43.3)	0.95 (0.86; 1.05)
Total cholesterol	mmol/L	5.81 (5.76–5.86)	6.03 (6.01–6.05)	**0.85 (0.81; 0.88) per 1 mmol/L**
HDL cholesterol	mmol/L	1.51 (1.49–1.53)	1.52 (1.51–1.53)	**1.13 (1.00; 1.27) per 1 mmol/L**
Subclinical inflammation	%	27.1 (25.3–28.9)	28.1 (27.2–29.0)	1.02 (0.92; 1.14)
Serum creatinine	nmol/L	76.2 (75.5–77.0)	76.4 (76.0–76.8)	1.00 (0.98; 1.03) per 10 nmol/L
Albuminuria	%	15.2 (13.6–17.0)	13.3 (12.5–14.1)	0.93 (0.79; 1.10)
Hemoglobin	g/dL	14.13 (14.07–14.18)	14.16 (14.14–14.19)	1.06 (0.89; 1.25)^a^ per 1 g/dL
Biomarkers of iron deficiency^b^				
Ferritin	μg/L	210 (268)	152 (148)	**1.12 (1.06; 1.18) per 100 μg/L**
Transferrin saturation	%	27.5 (13.1)	26.0 (10.6)	**1.13 (1.03; 1.23) per 10 %**
Erythrocyte protoporphyrin	μmol/L	1.09 (1.17)	0.93 (0.42)	**1.25 (1.14; 1.38) per 0.5 μmol/L**
Hypertension				
No hypertension	%	47.0 (45.1–49.0)	47.0 (46.1–48.0)	Ref
Known hypertension or systolic blood pressure ≥140 to <160 mmHg	%	39.8 (37.9–41.7)	40.2 (39.3–41.1)	1.02 (0.96; 1.10)
Systolic blood pressure ≥160 mmHg	%	14.2 (12.9–15.7)	14.0 (13.4–14.7)	1.03 (0.94; 1.13)
History of MI or stroke	%	7.9 (6.9–9.1)	7.1 (6.6–7.6)	1.04 (0.87; 1.24)
Biomarkers of liver function^b^				
GGT	U/L	44.3 (92.9)	29.9 (40.3)	**1.02 (1.00; 1.04) per 10 U/L**
AST	U/L	24.7 (18.7)	22.0 (11.9)	**1.12 (1.00; 1.26) per 10 U/L**
ALT	U/L	16.0 (13.4)	14.6 (11.3)	**0.86 (0.75; 0.996) per 10 U/L**

The table shows pooled means or proportions of baseline characteristics in the HbA_1c_ categories and additionally the results of a multivariable logistic regression model including all variables of the table as explanatory variables for very low HbA_1c_ (reference: low HbA_1c_). Bold indicates statistically significant difference (*p* <0.05). ^a^Random effects model reported because of statistically significant heterogeneity; ^b^assessed in NHANES, only. ALT, alanine transferase; AST, aspartate transferase; BMI, body mass index; GGT, gamma-glutamyl transferase; CI, confidence interval; HbA_1c_, glycated hemoglobin; HDL, high-density lipoprotein; MI, myocardial infarction

For being plausible explanations for an association of very low HbA_1c_ levels with mortality or cardiovascular outcomes, known cardiovascular/mortality risk factors should be associated with very low HbA_1c_ levels in the logistic regression model and with mortality outcomes in the same direction (i.e. being a risk factor for both very low HbA_1c_ and mortality). Risk factors of interest were underweight, subclinical inflammation, blood hemoglobin concentration (biomarker of anemia), ferritin, transferrin saturation or erythrocyte protoporphyrin (biomarkers of iron deficiency), alcohol consumption, albuminuria (biomarker of renal function), serum creatinine (biomarker of renal function), AST, ALT or GGT (biomarkers of liver function). Furthermore, adding the variables to an age- and sex-adjusted model for mortality should attenuate the observed association of very low HbA_1c_ with the outcome. The described analyses to identify plausible explanatory variables were only conducted in the NHANES because only this cohort assessed all variables of interest listed above.

For longitudinal analyses, Cox proportional hazards regression models were utilized after the proportional hazards assumption was tested with Schoenfeld residuals (which was fulfilled). We compared HbA_1c_ categories with respect to the outcomes all-cause mortality, cardiovascular mortality and primary cardiovascular events in a “simple”, age- and sex-adjusted model and the “full” model (see list of variables above, except no adjustment for history of MI or stroke for the outcome “primary cardiovascular events” because of exclusions).

We used a two-step approach: we first analyzed the single studies and pooled the results thereafter by meta-analysis. Meta-analyses were conducted with the statistical software Comprehensive Meta-Analysis 2.0 (Biostat, Englewood, NJ, USA). A one-step approach was not possible because UiT The Arctic University of Norway did not consent to send individual data of the Tromsø study to the analyzing center in Heidelberg, Germany. This was also the reason why a dose-response analysis with restricted cubic splines could not be conducted in a pooled data set. Instead, such curves were exemplarily retrieved from the NHANES with five a priori defined knots at HbA_1c_ levels of 4.5 %, 5 %, 5.5 % and 6.25 % and 5.25 % as the reference [35]. Results from the NHANES are a good approximation for the results from the total consortium because the NHANES assessed all potential confounders and dominated the meta-analyses with its high case numbers.

In meta-analyses, statistical heterogeneity among the studies was examined with Cochrane’s Q test and the I^2^ statistic. Fixed effects models were reported unless significant heterogeneity was present, taking only the sample size of the cohorts into account. In the few occasions of significant heterogeneity, this was indicated and random effects model results were reported, taking the sample size of the cohorts and the between-study variance into account. In the fixed effects model, the weight of the studies was expressed as the inverse of the variance of the logarithm of the estimated hazard ratio (HR). In the random effects model, a variation of the inverse-variance method, invented by DerSimonian and Laird, was applied, which adjusts for the heterogeneity in the meta-analysis and produces less precise pooled effect estimates than the fixed effects model [36].

Subgroup analyses were carried out for both sexes and two age-groups (<65 and ≥65 years). Subgroup analyses were restricted to cohorts that could contribute to subgroups with a sufficient number of events. In sensitivity analyses, cohorts with diagnoses of non-fatal events based on self-reported physician diagnoses (ELSA and SHIP) were excluded from analyses of primary cardiovascular events. If not stated otherwise, analyses were conducted with SAS 9.3 (SAS Institute Inc., Cary, NC, USA).

Multiple imputation was employed to impute the number of missing baseline covariate values shown in Additional file 1: Table S2. The proportion of missing values was below 5 % for most variables, between 5 % and 15 % on seven occasions and higher than 15 % on three occasions (HDL cholesterol in ESTHER (37.9 %), alcohol consumption in Tromsø (23.4 %) and GGT in the NHANES (25.9 %)). To the best of our knowledge, data were missing at random, which was the assumption of the multiple imputation. Separately for the cohorts, 20 complete data sets were imputed with the SAS 9.3 procedure “PROC MI”, using the Markov chain Monte Carlo method. Variables from the “full” model were used for the imputation model but the outcomes were not included. Variables were modelled continuously if possible and the logarithm was taken if they were not normally distributed (employed for BMI, systolic blood pressure, HDL cholesterol, CRP, urinary albumin, serum creatinine and GGT). This log-transformation of variables was applied in the multiple imputation process only. All multivariable analyses were performed in the 20 imputed data sets and results of the individual data sets were combined by the SAS 9.3 procedure “PROC MIANALYZE”, taking the variation between the results of the imputed data sets into account.

## Results

The baseline characteristics of the included subjects without diabetes mellitus from participating cohorts are shown in Table 1. All cohorts included an almost equal share of men and women and mean ages were between 61.6 and 67.9 years. With few exceptions, the baseline characteristics were similar in the cohorts. Furthermore, the mean HbA_1c_ was comparable across cohorts with values between 5.4 % (36 mmol/mol) and 5.6 % (38 mmol/mol). However, proportions of study participants with very low and increased HbA_1c_ levels varied substantially between the cohorts. Baseline characteristics of individuals with very low, intermediate and increased HbA_1c_ were compared with the reference group with low HbA_1c_ (see Table 2, Additional file 1: Table S3 and Table 3, respectively). The tables show pooled means or proportions of baseline characteristics in the HbA_1c_ categories and additionally the results of the multivariable logistic regression model including all variables from the respective table as explanatory variables for the HbA_1c_ difference. Very low HbA_1c_ levels were, in general, significantly positively associated with variables that indicated a good prognosis for cardiovascular outcomes and mortality; i.e. younger age, less frequent obesity, less frequent current smoking, lower total cholesterol and higher HDL cholesterol (Table 2). From those variables that could potentially explain an association of very low HbA_1c_ levels with cardiovascular outcomes and mortality, the following variables were not associated with very low HbA_1c_ levels: underweight; subclinical inflammation; low blood hemoglobin values; albuminuria; and high serum creatinine levels. However, from the potentially explanatory variables, the following variables were significantly associated with very low HbA_1c_ levels: non-Hispanic black race/ethnicity; high alcohol consumption; all three biomarkers of iron deficiency; and all three biomarkers of liver function.

**Table 3:** Baseline characteristics of subjects without diabetes mellitus by increased and low HbA_1c_ levels and multivariable adjusted odds ratios for associations of characteristics with increased HbA_1c_ levels. Pooled data from six cohort studies

Characteristic	Unit	Weighted mean or proportion (%) (95 % CI)		Pooled odds ratio (95 % CI)
		Increased HbA_1c_	Low HbA_1c_	
		(6.0 to <6.5 %)	(5.0 to <5.5 %)	
		(42 to <48 mmol/mol)	(31 to <37 mmol/mol) (reference group)	
Age	Years	64.5 (64.2–64.7)	62.2 (62.1–62.4)	**1.41 (1.32; 1.49) per 10 years**
Male sex	%	47.0 (45.2–48.7)	45.0 (44.1–46.0)	0.99 (0.88; 1.11)
Race/ethnicity^b^				
Non-Hispanic white	%	44.0	70.0	Ref
Non-Hispanic black	%	33.8	11.8	**5.92 (4.56; 7.68)**
Mexican-American	%	17.8	15.1	**2.11 (1.62; 2.77)**
Other	%	4.4	3.0	**2.69 (1.69; 4.28)**
School education				
≤9 years	%	55.6 (53.7–57.5)	45.9 (44.9–46.9)	Ref
10–12 years	%	32.7 (30.9–34.4)	39.1 (38.1–40.0)	0.97 (0.90; 1.04)
≥13 years	%	14.6 (13.3–16.0)	19.0 (18.3–19.8)	0.99 (0.90; 1.08)
BMI category				
Underweight or low weight	%	3.2 (2.5–3.9)	3.9 (3.5–4.3)	**0.81 (0.66; 0.99)**
Optimal weight	%	20.3 (18.9–21.8)	34.6 (33.7–35.5)	Ref
Overweight	%	45.3 (43.5–47.0)	43.6 (42.7–44.6)	1.02 (0.92; 1.14)
Obese	%	32.4 (30.8–34.1)	19.2 (18.5–20.0)	**1.61 (1.43; 1.81)**
Smoking				
Never	%	44.5 (42.7–46.3)	45.8 (44.8–46.7)	Ref
Former	%	31.9 (30.2–33.5)	34.8 (33.9–35.7)	1.03 (0.92; 1.16)
Current	%	24.1 (22.6–25.7)	20.0 (19.5–21.1)	**1.73 (1.53; 1.96)**
Relative alcohol consumption				
Abstainer or low	%	62.9 (61.1–64.7)	50.9 (49.9–51.9)	Ref
Moderate	%	30.5 (28.8–32.2)	37.4 (36.4–38.3)	1.05 (0.96; 1.13)
High	%	6.9 (6.0–7.9)	12.0 (11.4–12.7)	**0.74 (0.66; 0.83)**
Vigorous physical activity	%	36.0 (34.3–37.7)	42.3 (41.4–43.3)	0.93 (0.85; 1.02)
Total cholesterol	mmol/L	6.08 (6.03–6.12)	6.03 (6.01–6.05)	**1.18 (1.14; 1.22) per 1 mmol/L**
HDL cholesterol	mmol/L	1.36 (1.34–1.37)	1.52 (1.51–1.53)	**0.62 (0.54; 0.71) per 1 mmol/L**
Subclinical inflammation	%	44.7 (42.9–46.4)	28.1 (27.2–29.0)	**1.47 (1.34; 1.61)**
Serum creatinine	nmol/L	85.4 (84.5–86.3)	76.4 (76.0–76.8)	**1.04 (1.00; 1.08)** ^**a**^ **per 10 nmol/L**
Albuminuria	%	21.2 (19.6–23.0)	13.3 (12.5–14.1)	**1.27 (1.11; 1.45)**
Hemoglobin	g/dL	13.98 (13.92–14.04)	14.16 (14.14–14.19)	**0.86 (0.77; 0.96)** ^**a**^ **per 1 g/dL**
Biomarkers of iron deficiency^b^				
Ferritin	μg/L	165 (170)	152 (148)	0.97 (0.91; 1.03) per 100 μg/L
Transferrin saturation	%	23.8 (10.1)	26.0 (10.6)	**0.85 (0.77; 0.95) per 10 %**
Erythrocyte protoporphyrin	μmol/L	0.94 (0.44)	0.93 (0.42)	1.02 (0.91; 1.15) per 0.5 μmol/L
Hypertension				
No hypertension	%	34.7 (33.1–36.4)	47.0 (46.1–48.0)	Ref
Known hypertension or systolic blood pressure ≥140 to <160 mmHg	%	49.5 (47.8–51.3)	40.2 (39.3–41.1)	**1.08 (1.01; 1.15)**
Systolic blood pressure ≥160 mmHg	%	17.1 (15.8–18.5)	14.0 (13.4–14.7)	**1.10 (1.01; 1.20)**
History of MI or stroke	%	11.8 (10.7–13.0)	7.1 (6.6–7.6)	**1.32 (1.03; 1.70)** ^**a**^
Biomarkers of liver function^b^				
GGT	U/L	33.5 (36.0)	29.9 (40.3)	1.02 (0.99; 1.05) per 10 U/L
AST	U/L	21.5 (13.1)	22.0 (11.9)	**0.81 (0.70; 0.94) per 10 U/L**
ALT	U/L	15.0 (11.2)	14.6 (11.3)	**1.25 (1.08; 1.45) per 10 U/L**

The table shows pooled means or proportions of baseline characteristics in the HbA_1c_ categories and additionally the results of a multivariable logistic regression model including all variables of the table as explanatory variables for increased HbA_1c_ (reference: low HbA_1c_). Bold indicates statistically significant difference (*p* <0.05). ^a^Random effects model reported because of statistically significant heterogeneity; ^b^assessed in NHANES, only. ALT, alanine transferase; AST, aspartate transferase; BMI, body mass index; GGT, gamma-glutamyl transferase; CI, confidence interval; HbA_1c_, glycated hemoglobin; HDL, high-density lipoprotein; MI, myocardial infarction

In contrast to the results for very low HbA_1c_, increased HbA_1c_ levels were generally significantly associated with characteristics that are associated with adverse cardiovascular outcomes and mortality; i.e. older age, other than non-Hispanic white race/ethnicity, obesity, current smoking, high total cholesterol, low HDL cholesterol, subclinical inflammation, high serum creatinine, albuminuria, low blood hemoglobin concentration, low transferrin saturation, hypertension, a history of MI or stroke and high ALT, with the exceptions of high alcohol consumption, underweight, high GGT and high AST (Table 3). Results for the intermediate group point in the same direction for these variables as outlined for the group with increased HbA_1c_ but with lower effect estimates (Additional file 1: Table S3). Overall, a consistent step-wise increase in the burden of cardiovascular and mortality risk factors was observed with increasing HbA_1c_ levels in subjects without diabetes mellitus. The exceptions were high alcohol consumption associated with a higher proportion in subjects with very low HbA_1c_ levels (15.7 %) than in subjects with low (12.0 %), intermediate (9.0 %) and increased HbA_1c_ (6.9 %), the biomarkers of liver function (GGT, AST, ALT) and ferritin levels with highest values in subjects with very low HbA_1c_ levels and second highest values in subjects with increased HbA_1c_ (U-shaped associations) as well as erythrocyte protoporphyrin levels, which were increased in the very low HbA_1c_ group, only.

For the longitudinal analyses, the mean follow-up time varied by cohort and outcome between 5.3 and 14.1 years (Table 1, bottom). In summary, 6,769 of 28,681 study participants died during a mean follow-up of 10.7 years (standard deviation (SD) 3.6) of whom 2,648 died of cardiovascular disease. Of those 20,490 study participants without a history of MI or stroke at baseline, 2,493 experienced a primary cardiovascular event during a mean follow-up of 8.5 years.

**Fig. 1:**
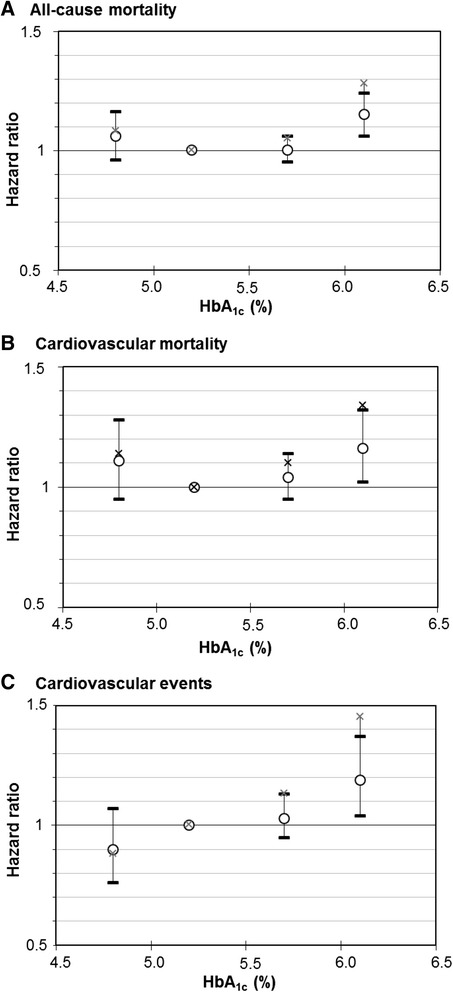
Dose-response relationship of meta-analyzed associations of HbA_1c_ levels with (**a**) all-cause mortality, (**b**) cardiovascular mortality and (**c**) cardiovascular outcomes in subjects without diabetes mellitus with increasing adjustment for potential confounders. Crosses, point estimates of “simple” model; circles with 95 % confidence intervals, effect estimates of “full” model. Reference group: HbA_1c_ 5.0 to <5.5 % (31 to <37 mmol/mol)

Figure 1 shows the dose-response relationship of HbA_1c_ levels with these outcomes for the “simple” model (only age- and sex-adjusted) and “full” model (adjusted for all variables of Table 1 except biomarkers of liver function and iron deficiency, which were only assessed in the NHANES). J-shaped dose-response curves are suggested by the observed effect estimates for all-cause and cardiovascular mortality, whereas the association of HbA_1c_ levels with cardiovascular events appears to be rather linear in both models. Hazard ratios (HR [95 % confidence interval]) of the “full” model for the comparison of subjects with very low to those with low HbA_1c_ levels were weak and not statistically significant for the outcomes all-cause mortality (1.06 [0.96; 1.16]; Additional file 1: Table S4), cardiovascular mortality (1.08 [0.79; 1.47]; Additional file 1: Table S5) and cardiovascular events (0.90 [0.76; 1.07]; Additional file 1: Table S6) and only slightly attenuated compared to the HRs of the “simple” model.

**Fig. 2:**
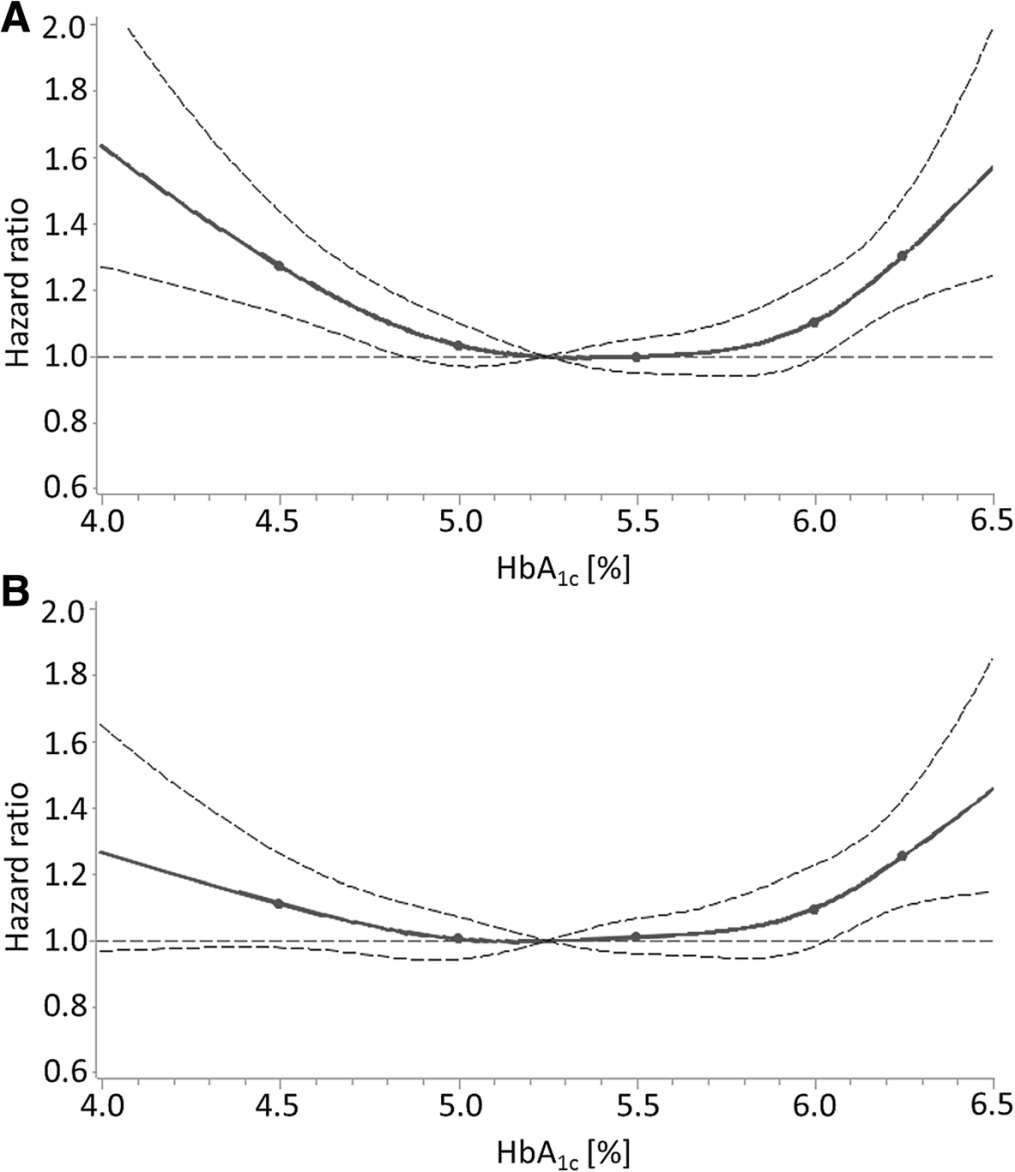
Dose-response relationship of HbA_1c_ levels with all-cause mortality in subjects without diabetes mellitus in the NHANES with (**a**) adjustment for age and sex and (**b**) adjustment for all potential confounders (“full” model including biomarkers of iron deficiency and liver function). Solid line, estimation; points in solid lines, knots; horizontal dashed line, null effect value (hazard ratio = 1); curved dashed lines, boundaries of 95 % confidence interval band of estimation

However, these effect estimates were not adjusted for biomarkers of liver function and iron deficiency, which were only available in the NHANES, and the impact of adjusting for these potential confounders can be seen for the outcome “all-cause mortality” in Fig. 2 (the same pattern was observed for the outcome “cardiovascular mortality” (Additional file 1: Figure S1)). The initially statistical significant association of HbA_1c_ levels <4.9 % with mortality was strongly attenuated and lost statistical significance.

As the next step, we aimed to identify all important confounders of the association of very low HbA_1c_ levels with mortality outcomes in the NHANES. To be able to explain an increased mortality risk at very low HbA_1c_ levels, the confounder needs to be associated with very low HbA_1c_ levels and mortality in the same direction (e.g. increases both risk for very low HbA_1c_ levels and mortality). The associations of all assessed variables with all-cause and cardiovascular mortality in the NHANES are shown in Additional file 1: Table S8. By comparing the directions of the effect estimates in Table 2 and Additional file 1: Table S8, 11 potential confounders were identified. Hemoglobin was exceptionally added because there was statistically significant heterogeneity in the meta-analysis and increasing hemoglobin concentration was significantly protective for very low HbA_1c_ levels in the NHANES (i.e. the same direction as for mortality). However, only the following eight variables actually attenuated the strength of the association of very low HbA_1c_ levels with both all-cause and cardiovascular mortality when added to an age- and sex-adjusted model (Table 4): race/ethnicity; alcohol consumption; BMI; hemoglobin; ferritin; erythrocyte protoporphyrin; GGT; and ALT. Hemoglobin was the strongest confounder but the other seven variables also contributed modestly to an overall strong attenuation of the effect estimates because the strength of the association increased again when they were dropped from the final model (data not shown). Adding further variables from Table 1 to the model did not lead to further attenuation, indicating that the main confounders of the association are included in this model. However, excluding non-Hispanic blacks from the analysis led to a further attenuation of the association for all-cause mortality (HR 1.048 [0.906; 1.211]) and cardiovascular mortality (HR 1.098 [0.885; 1.363]).

**Table 4:** Attenuation of strength of the association of very low HbA_1c_ levels (<5 % (<31 mmol/mol)) with all-cause and cardiovascular mortality by adding potential confounders to the “simple” model in the NHANES

Model	HR (95 % CI)^a^		Attenuation for both outcomes
	All-cause mortality	CV mortality	
Age + sex (“simple” model)	1.201 (1.057; 1.328)	1.221 (1.005; 1.409)	N/A
Age + sex + race/ethnicity	1.184 (1.042; 1.341)	1.203 (0.989; 1.453)	Yes
Age + sex + alcohol consumption	1.192 (1.051; 1.353)	1.212 (1.000; 1.469)	Yes
Age + sex + BMI	1.172 (1.033; 1.330)	1.197 (0.988; 1.450)	Yes
Age + sex + physical activity	1.208 (1.065; 1.370)	1.231 (1.016; 1.491)	No
Age + sex + hemoglobin	1.166 (1.027; 1.324)	1.188 (0.980; 1.440)	Yes
Age + sex + ferritin	1.193 (1.052; 1.354)	1.211 (0.999; 1.467)	Yes
Age + sex + total cholesterol	1.192 (1.051; 1.353)	1.226 (1.012; 1.486)	No
Age + sex + erythrocyte protoporphyrin	1.177 (1.036; 1.337)	1.208 (0.995; 1.466)	Yes
Age + sex + GGT	1.178 (1.038; 1.337)	1.206 (0.995; 1.462)	Yes
Age + sex + AST	1.199 (1.057; 1.361)	1.222 (1.009; 1.481)	No
Age + sex + ALT	1.199 (1.057; 1.360)	1.215 (1.003; 1.472)	Yes
Age + sex + race/ethnicity + alcohol consumption + BMI + ferritin + erythrocyte protoporphyrin + GGT + ALT	1.081 (0.950; 1.230)	1.106 (0.909; 1.345)	
All variables of Table 1	1.103 (0.968; 1.256)	1.120 (0.921; 1.363)	

^a^Reference group: low HbA_1c_ (5.0 to <5.5 % (31 to <37 mmol/mol)). ALT, alanine transferase; AST, aspartate transferase; BMI, body mass index; CV, cardiovascular; GGT, gamma-glutamyl transferase; HR, hazard ratio, N/A, not applicable

In the meta-analysis of all cohorts, HRs for the comparison of subjects with increased HbA_1c_ levels and subjects with low levels were strongly attenuated by increasing adjustment for cardiovascular risk factors but remained statistically significant: all-cause mortality (1.14 [1.03; 1.27]; Additional file 1: Table S4); cardiovascular mortality (1.17 [1.00; 1.37]; *P* <0.05; Additional file 1: Table S5); and cardiovascular events (1.19 [1.04; 1.37]; Additional file 1: Table S6). The covariates that were most responsible for the attenuations were smoking, CRP and the renal function biomarkers serum creatinine and albuminuria (data not shown).

No association of intermediate HbA_1c_ levels with any of the outcomes was observed in the “full” model (HR point estimates between 1.00 and HR 1.03; Additional file 1: Table S4–S6). Statistically significant heterogeneity was only observed in meta-analyses on very low HbA_1c_ levels and mortality outcomes with associations indicating a potentially increased mortality risk at very low HbA_1c_ levels (HR >1.10) in three cohorts, a potential protective effect (HR <0.90) in one cohort and potentially no effect (HR 0.90–1.10) in two cohorts (Additional file 1: Table S4).

An interaction term of the variables “very low versus low HbA_1c_” and “age <65 versus ≥65 years” was statistically significantly associated with the outcome “primary cardiovascular events” (*p* = 0.006) but not with all-cause mortality (*p* = 0.399) or cardiovascular mortality (*p* = 0.449). Results from meta-analyses of all cohorts stratified by age <65 years and age ≥65 years are shown in Table 5. Observed HRs for all-cause and cardiovascular mortality were 1.13 [1.01; 1.27] and 1.33 [0.86; 1.27], respectively, for subjects aged ≥65 years and 0.95 [0.81; 1.12] and 0.99 [0.74; 1.33], respectively, for individuals aged 50–64 years. A stronger age-difference was observed for the outcome “primary cardiovascular events” with an observed HR of 1.15 [0.90; 1.46] in subjects aged ≥65 years and 0.76 [0.60; 0.97] in subjects aged 50–64 years, the latter even showing a statistically significant protective association (Table 5). The potential age-difference was investigated in greater detail in a sensitivity analysis in the cohort of the consortium with the highest case numbers, the NHANES. Stratification in 5-year age-intervals in the NHANES did not show a clear pattern towards stronger associations in older age strata for the outcomes all-cause and cardiovascular mortality (Additional file 1: Table S9). The outcome “primary cardiovascular events” was unfortunately not assessed in the NHANES. No age differences were observed for the intermediate and increased HbA_1c_ category. Furthermore, no relevant sex differences were observed (Additional file 1: Table S7).

**Table 5:** Age-stratified analyses of the associations of HbA_1c_ levels with mortality and cardiovascular outcomes in subjects without diabetes mellitus

Outcome/stratum	Very low HbA_1c_ (<5.0 %) (<31 mmol/mol)				Low HbA_1c_ (5.0 to <5.5 %) (31 to <37 mmol/mol)				Intermediate HbA_1c_ (5.5 to <6.0 %) (37 to <42 mmol/mol)				Increased HbA_1c_ (6.0 to <6.5 %) (42 to <48 mmol/mol)			
	n_total_	n_cases_	IR^a^	HR (95 % CI)^b^	n_total_	n_cases_	IR^a^	HR	n_total_	n_cases_	IR^a^	HR (95 % CI)^b^	n_total_	n_cases_	IR^a^	HR (95 % CI)^b^
All-cause mortality																
50–64 years	1,688	205	10.0	0.95 (0.81; 1.12)	6,563	769	9.8	Ref	6,336	761	10.1	0.91 (0.82; 1.01)	1,514	272	15.8	**1.18 (1.02; 1.37)**
≥65 years	632	314	53.3	**1.13 (1.01; 1.27)**	3,779	1,556	42.6	Ref	4,767	1,978	43.3	1.06 (0.99; 1.13)	1,413	723	50.4	**1.14 (1.04; 1.26)**
Cardiovascular mortality																
50–64 years	1,218	61	3.7	0.99 (0.74; 1.33)	4,824	210	3.2	Ref	4,915	220	3.4	0.93 (0.76; 1.13)	1,194	78	5.3	1.14 (0.87; 1.50)
≥65 years	548	155	28.4	1.33 (0.86; 2.04)^c^	2,574	589	20.9	Ref	3,343	753	21.2	1.07 (0.82; 1.39)^c^ ^d^	1,035	249	24.2	**1.18 (1.01; 1.38)**
Cardiovascular events																
50–64 years	1,276	77	5.1	**0.76 (0.60; 0.97)**	5,626	456	8.1	Ref	5,470	507	9.6	1.09 (0.96; 1.25)	1,165	114	10.6	1.18 (0.95; 1.47)
≥65 years	409	82	22.4	1.15 (0.90; 1.46)	2,491	488	22.8	Ref	3,207	598	23.0	0.99 (0.88; 1.12)	846	171	26.7	1.19 (0.98; 1.45)

Bold indicates statistically significant difference (*p* <0.05). ^a^IR, incidence rate per 1,000 person-years. The IR is the weighted mean of the IRs of the individual studies (weighted by sample size); ^b^adjusted for age, sex, race/ethnicity, BMI, education, smoking, physical activity, alcohol consumption, total cholesterol, HDL cholesterol, CRP, hemoglobin, serum creatinine, albuminuria, hypertension and history of CVD; ^c^random effects model result reported due to statistical significant heterogeneity. CI, confidence interval; HbA_1c_, glycated hemoglobin; HR, hazard ratio; IR, incidence rate; n, sample size

Results also did not change when biomarkers of liver function were modelled in quintiles or dichotomously based on clinical cut-points indicating potential liver disease (data not shown). Finally, excluding cohorts with self-reported MI or stroke information from analyses did not change the overall results (data not shown).

## Discussion

In this individual participant data meta-analysis of six prospective cohort studies in subjects without diabetes mellitus, a linear association of HbA_1c_ levels with primary cardiovascular events was observed. The observed effect estimates for increased HbA_1c_ levels (6.0 to <6.5 % (42 to <48 mmol/mol)) were strongly attenuated by adjustment for cardiovascular risk factors (mainly by adjustment for smoking, inflammatory status and renal function) but remained statistically significant for all three outcomes (primary cardiovascular events, all-cause mortality and cardiovascular mortality). At the lower end of HbA_1c_ levels, cohorts of the consortium yielded inconsistent results for the mortality outcomes and the pooled effect estimate was not statistically significant. In one cohort with a pronounced J-shaped association of HbA_1c_ levels with all-cause and cardiovascular mortality (NHANES), the following confounders of the association of very low HbA_1c_ levels with mortality outcomes were identified: race/ethnicity; alcohol consumption; BMI; as well as biomarkers of iron deficiency anemia and liver function. The association of very low HbA_1c_ levels with mortality outcomes also lost statistical significance in this cohort after adjusting for these confounders.

The observed increased cardiovascular risk and increased mortality of subjects without diabetes mellitus but with increased HbA_1c_ (6.0 to <6.5 % (42 to <48 mmol/mol)) is in agreement with results from previous population-based cohort studies [5, 6, 11, 12, 16–18, 37–40]. This consistent finding from observational studies is supported by the fact that coronary atherosclerosis and plaque vulnerability are advanced in subjects with increased HbA_1c_ levels even if they are below the threshold for a diabetes diagnosis [41]. However, we observed a strong attenuation of effect estimates by adjustment for conventional cardiovascular risk factors, which was also observed by others [5–7]. This attenuation could be explained by confounding mostly by smoking, CRP and renal function, factors which were associated with increased HbA_1c_. The fact that smokers have higher HbA_1c_ levels than non-smokers or ex-smokers was also observed in another consortium of cohort studies [42]. Nevertheless, effect estimates remained statistically significant in comprehensively adjusted models, which could indicate a small independent contribution of impaired glucose metabolism, below the diagnostic threshold for diabetes mellitus, to the development of cardiovascular disease. However, the small effect estimates could also be simply due to residual confounding because it is impossible to perfectly adjust for all cardiovascular risk factors in observational studies. The majority of randomized controlled trials (RCTs) in non-diabetic subjects with increased HbA_1c_ failed to observe significant effects when aiming to reduce the cardiovascular risk and mortality of these individuals [43]. However, the short average follow-up time of 3.75 years was a limitation of previous trials and further RCTs, with larger sample size and longer follow-up are required to explore the efficacy of non-drug and drug-based approaches to reduce the cardiovascular risk of non-diabetic subjects with increased HbA_1c_ [43].

With respect to very low HbA_1c_ in subjects without diabetes mellitus, this meta-analysis of the CHANCES consortium yielded inconsistent results for the outcomes all-cause and cardiovascular mortality and a consistent, albeit statistically non-significant decreased risk for primary cardiovascular events in subjects with an HbA_1c_ <5 % (<31 mmol/mol). The latter contrasts with the meta-analysis of the ERFC that observed a significantly increased cardiovascular risk in subjects with an HbA_1c_ <4.5 % (<26 mmol/mol) (HR 1.23 [1.02; 1.50]) compared with subjects with an HbA_1c_ of 5 to <5.5 % (31 to <37 mmol/mol). However, the result for this HbA_1c_ category was only based on 127 cardiovascular events from 24 studies. Our meta-analysis included 159 cardiovascular events from five studies in the lowest HbA_1c_ category (<5 % (<31 mmol/mol)). Low numbers of events from single studies can affect the model stability and can result in high point estimates with wide confidence intervals. Therefore, despite the overall large sample size of this meta-analysis and the meta-analysis of the ERFC, the results for the lowest HbA_1c_ category of both could be biased by low sample sizes and be random findings.

Because many previous studies [13–18] have observed an increased mortality of non-diabetics with very low HbA_1c_ levels and this was also found in two out of six of the cohorts included in our meta-analysis (NHANES and ESTHER), we aimed to explore potential explanations in the NHANES because this study assessed all relevant variables that could confound the association of very low HbA_1c_ levels and mortality. The strongest confounder was anemia, which was expected because hemoglobin and HbA_1c_ concentrations are correlated [32]. The biomarkers of iron deficiency ferritin and erythrocyte protoporphyrin were also identified as confounders but whether iron-deficiency anemia, non-iron-deficiency anemia or both are of relevance for very low HbA_1c_ levels needs to be determined by further studies because the underlying biology is complex [21]. Non-Hispanic black race/ethnicity was also an expected confounder because African-Americans, compared with white Americans, have a different hematologic profile including lower hemoglobin values [26]. High alcohol consumption and biomarkers of liver disease were further confounders, which could be explained by an inhibition of the gluconeogenesis in the liver [23] and a shortening the red blood cell lifespan. Very low HbA_1c_ values can simply originate from everything that reduces the red blood cell lifespan because some time is needed for glucose and hemoglobin to interact and form glycosylated hemoglobin [24]. BMI also played a role but not as expected. Underweight was not associated with very low HbA_1c_ levels and obesity was protective for very low HbA_1c_ levels. The confounding for mortality could result from obesity that has been found to be protective for mortality at older age [44]. However, it is yet unclear whether this “obesity paradox” is due to statistical biases or has a plausible underlying biology [44]. In summary, from the hypotheses listed in the introduction, only subclinical inflammation and renal function were not confirmed as confounders for the association of very low HbA_1c_ levels and mortality in the NHANES.

The first and, to our knowledge, only other study that aimed to discover potential mechanisms that could explain an increased risk of mortality in subjects with very low HbA_1c_ levels did not find any attenuation of the strength of the association of very low HbA_1c_ levels (<5.0 % (<31 mmol/mol)) with all-cause mortality after additional adjustment for diseases, weight measures, education, alcohol consumption, physical activity, smoking, hemoglobin, red blood cell mean corpuscular volume, fibrinogen and leukocyte count [17].

In our meta-analysis, a significant interaction was observed between very low HbA_1c_ levels and age for the outcome “primary cardiovascular events”. To our knowledge, this is a novel finding but since it is from a subgroup analysis, further studies are required to corroborate this interaction with age. There is room for doubts, because this interaction was not significant for the other outcomes “all-cause mortality” and “cardiovascular mortality” and stratification by 5-year intervals in the NHANES also showed that there was no age-difference in the association of very low HbA_1c_ levels with fatal outcomes.

When interpreting our results, the following limitations and strengths should be considered. The main limitation of this meta-analysis is the observational nature of the included prospective cohort studies. Despite adjustment for known potential confounders, we cannot rule out the possibility that the observed associations are confounded by other unmeasured factors (e.g. biomarkers of liver function and iron deficiency in cohorts other than NHANES and other unmeasured factors known to affect HbA_1c_ assay test results, such as participation in endurance sport, late pregnancy and major blood loss [45]) or residual confounding by variables that could have been more precisely measured (e.g. physical activity). It can be expected that some residual confounding is present in the data and observed small effect sizes for increased HbA_1c_ levels could be due to residual confounding despite the observed statistical significant associations. Furthermore, it is possible that people with pre-diabetes at baseline developed manifest diabetes mellitus in the first years of follow-up and experienced a cardiovascular outcome or death in later follow-up due to diabetes and not pre-diabetes. However, diabetes incidence information was not collected for this analysis and this could not be further elucidated.

Other limitations include the fact that non-fatal MI and stroke information was solely based on self-reported information in two cohorts but the overall results did not change when these cohorts were excluded in sensitivity analysis. Furthermore, other glucose measures (i.e. fasting glucose or measures based on an oral glucose tolerance test) were not included, which could have yielded different results. Furthermore, HbA_1c_ was measured once whereas measurements at different time-points could have corrected better for intra-individual variation and random measurement errors. In addition, different HbA_1c_ assays were applied in the cohorts but they were all traceable to the assay of the DCCT trial and therefore comparable.

Strengths of our study include the variety of cohorts from all over Europe and the United States, the overall large size enabling subgroup analyses for age and sex, almost complete mortality registry-based follow-ups and the common statistical analysis strategy. A particular advantage over previous studies is the adjustment for a large number of cardiovascular risk factors including biomarkers of inflammation, renal function, lipid metabolism, liver function and anemia.

## Conclusions

In this meta-analysis of subjects without diabetes mellitus from six prospective cohort studies a linear association of HbA_1c_ levels with primary cardiovascular events was observed. The observed effect estimates for increased HbA_1c_ levels were strongly attenuated by adjustment for cardiovascular risk factors for all three outcomes (primary cardiovascular events, all-cause mortality and cardiovascular mortality). The cohorts yielded inconsistent results for the associations of very low HbA_1c_ levels with mortality outcomes. For cardiovascular and all-cause mortality, the observed small effect sizes at both the lower and upper end of HbA_1c_ distribution do not support the notion of a J-shaped association of HbA_1c_ levels because a certain degree of residual confounding was likely present in the meta-analyses, which could not be adjusted for iron deficiency anemia and liver function as they were not assessed in most cohorts.

### Availability of data and materials

No data will be shared because of data protection regulations.

## Additional file

Additional file 1:
**Cohort descriptions, Table S1–S9 and Figure S1.** (DOC 450 kb)
