# Supplementary material for: HbA1c levels in non-diabetic older adults – No J-shaped associations with primary cardiovascular events, cardiovascular and all-cause mortality after adjustment for confounders in a meta-analysis of individual participant data from six cohort studies
Source: BMC Med. 2016 Feb 11;14:26. doi: 10.1186/s12916-016-0570-1 (PMC4751667; doi:10.1186/s12916-016-0570-1)
Supplement: Additional file 1: — Cohort descriptions, Table S1–S9 and Figure S1. (DOC 450 kb) [file 12916_2016_570_MOESM1_ESM.doc]

**Supplemental Material**

Table of Contents

[Description of included studies 2](#__RefHeading___Toc395778819)

[Suppl. Table S1 – Source of information for selected baseline covariates 6](#__RefHeading___Toc395778820)

[Suppl. Table S2 – Proportion of missing values in analyzed samples from the six population- based cohorts 7](#__RefHeading___Toc395778821)

[Suppl. Table S3 – Baseline characteristics of subjects without diabetes mellitus by intermediate and low HbA_1c_ levels, and multivariable adjusted odds ratios for associations of characteristics with intermediate HbA_1c_ levels. Pooled data from six cohort studies. 8](#__RefHeading___Toc395778822)

[Suppl. Table S4 – Associations of HbA_1c_ levels with all-cause mortality in subjects without diabetes mellitus 10](#__RefHeading___Toc395778823)

Suppl. Table S5 – Associations of HbA_1c_ levels with cardiovascular mortality in subjects without diabetes mellitus………………………………………………………………..……11

Suppl. Table S6 – Associations of HbA_1c_ levels with cardiovascular events in subjects without diabetes mellitus………………………………………………………………….….12

Suppl. Table S7 – Sex stratified analyses of the associations of HbA_1c_ levels with mortality and cardiovascular outcomes in subjects without diabetes mellitus..…………….…13

Suppl. Table S8 – Associations of potential confounders with all-cause mortality and cardiovascular mortality in subjects without diabetes mellitus in the NHANES........................................................................................................... 14

Suppl. Table S9 – Association of very low HbA_1c_ levels (<5%) with all-cause mortality in subjects without diabetes mellitus from NHANES stratified by 5-year age groups (comparison group: Low HbA_1c_ (5.0-5.5%)).......................................................16

Suppl. Figure 1 – Dose-response relationship of HbA_1c_ levels with cardiovascular mortality in subjects without diabetes mellitus in the NHANES with (A) adjustment for age and sex and (B) adjustment for all potential confounders (full model) ……..….17

Further funding sources of participating cohorts ……………………………………………………….18

# Description of included studies

**ELSA**

**Recruitment and data collection:** The **E**nglish **L**ongitudinal **S**tudy of **A**geing (ELSA) is a large, nationally representative study using a panel of individuals aged 50 years and over. ELSA is aimed at gaining a deeper understanding of the physical, psychological, social and economic aspects of ageing. The first wave of ELSA was carried out in 2002 and over 12,000 core sample members and their partners were interviewed during this wave. Participants are interviewed every two years and a nurse visit is carried out at every other wave. Wave 2 (2004/2005) was used as a baseline for this analysis because HbA_1c_ measurements were carried out for the first time in this wave.

**Ethics committee(s) that approved the study:** Ethical approval for all the ELSA waves was granted from the National Research and Ethics Committee.

**HbA_1c_ assay information:** Measurement of glycated haemoglobin was carried out by the Haematology Department at Royal Victoria Infirmary (Newcastle) using the Tosoh G7.

**Mortality follow-up:** Deaths that occurred until 31^st^ March 2012 were inquired at a nation-wide mortality registry.

**Non-fatal mon-fatal myocardial infarction and stroke notification source and procedure:** Non-fatal incident myocardial infarction and stroke events were self-reported in wave 3 (05/2006-08/2007), 4 (05/2008-07/2009) and 5 (06/2010-07/2012).

**ESTHER**

**Recruitment and data collection:** The “***E****pidemiologische* ***S****tudie zu Chancen der Verhütung, Früherkennung und optimierten* ***Th****erapie chronischer* ***Er****krankungen in der älteren Bevölkerung* [German]” (ESTHER) study is a population-based cohort of 9949 adults, aged 50-74 years at baseline, recruited by their general practitioners during a routine health check-up between 2000 and 2002 in the German federal state Saarland. At baseline, participants completed a comprehensive self-administered questionnaire, medical data and biological samples (blood, stool, urine) were collected and stored at -80°C for further analyses.

**Ethics committee(s) that approved the study:** Ethics committees of the Medical Faculty of the University of Heidelberg and the Medical Association of Saarland.

**HbA_1c_ assay information:** HbA_1c_ was measured at the central laboratory of the University Clinic of Heidelberg by High Performance Liquid Chromatography (HPLC) with the Bio-Rad Variant II (Bio-Rad, Munich, Germany).

**Mortality follow-up:** Deaths during follow-up until Dec 31, 2012 were identified by inquiry at the residents’ registration offices and death certificates were provided by public health departments.

**Non-fatal myocardial infarction and stroke notification source and procedure:** After 2, 5, 8 and 11 years of follow-up, study participants completed standardized questionnaires. Self-reported cases were validated by questionnaires sent to physicians.

**KORA**

**Recruitment and data collection:** The ***Ko****operative Gesundheitsforschung in der* ***R****egion* ***A****ugsburg* [Cooperative Health Research in the Region of Augsburg] (KORA) consists of four independent population-based surveys (S). The first three surveys were conducted as part of the World Health Organization (WHO) **Moni**toring of Trends and Determinants in **Ca**rdiovascular Disease (MONICA) project in 1984–1985 (S1), 1989–1990 (S2) and in 1994–1995 (S3). The fourth survey was conducted as part of KORA in 1999-2001 (S4). An HbA_1c_ assay traceable to the standard of the Diabetes Control and Complications Trial was applied in the S4 round and therefore only this round was used in this analysis. In Survey 4, subjects were invited to participate from the city of Augsburg and 16 towns and villages out of 70 communities from the surrounding districts with about 600,000 inhabitants in 1999-2001. Within each selected community, a stratified sample with ten equal strata by sex and age (range 25 to 74 years) was drawn. In total, 4261 subjects were recruited in the S4 round.

**Ethics committee(s) that approved the study:** Ethics Committee of the Bavarian Medical Association.

**HbA_1c_ assay information:** HbA_1c_-values were assessed at the laboratory of Augsburg Central

Hospital using a turbidimetric immunologic assay (Tina-quant, Roche Diagnostics).

**Mortality follow-up:** If a person did not return the follow-up questionnaire, the person's vital status was ascertained through the population registries inside and outside the study area. If the person died, the information on when and where is provided by the population registries and a copy of the death certificate is obtained from the Regional Health Department.

**Non-fatal myocardial infarction and stroke notification source and procedure:** Follow-up questionnaires were sent to each former participant in 2002-2003 and in 2008-2009 to obtain information on the occurrence of chronic diseases. Self-reported incident cases of myocardial infarction or stroke and the date of diagnosis were validated by hospital records or by contacting the treating physician. Furthermore, each time a non-fatal event was reported to the Coronary Events Registry, it was linked to the cohorts using name and date of birth.

**NHANES**

**Recruitment and data collection:** The **N**ational **H**ealth **a**nd **N**utrition **E**xamination **S**urvey (NHANES) is a program of studies designed to assess the health and nutritional status of adults and children in the United States. The latter is a cross-sectional study conducted by the National Center for Health Statistics (NCHS) between 1988 and 1994 and was designed as a multistage stratified, clustered probability sample of the US civilian non-institutionalized population at least two months old. The included subjects participated in an interview conducted at home and an extensive physical examination. In total, 30,818 people were interviewed in NHANES III (1997) and had a physical examination and a blood sample taken.

**Ethics committee(s) that approved the study:** National Center for Health Statistics (NCHS) Research Ethics Review Board.

**HbA_1c_ assay information:** HbA_1c_ measurements were performed by the Diabetes Diagnostic Laboratory at the University of Missouri (Columbia) using the Diamat Analyzer System (Bio-Rad Laboratories, Hercules, CA).

**Mortality follow-up:** A mortality linkage of NHANES III to death certificate data obtained in the National Death Index until Dec 31, 2006 was established.

**Non-fatal myocardial infarction and stroke notification source and procedure:** Not available.

**SHIP**

**Recruitment and data collection:** The **S**tudy of **H**ealth **i**n **P**omerania (SHIP) is a population-based study. The SHIP investigates common risk factors, subclinical disorders and manifest diseases in northeast Germany. From the total population of West Pomerania comprising 213 057 inhabitants in 1996, a two-stage stratified cluster sample of adults aged 20–79 years was drawn. In total, 4,305 subjects participated in the baseline examinations performed between 1997 and 2001 (SHIP-0).

**Ethics committee(s) that approved the study:** Ethics committees of the Medical Faculty of the University of Greifswald and of the medical chamber of Mecklenburg-Vorpommern

**HbA_1c_ assay information:** HbA_1c_ levels were measured by HPLC with the Bio-Rad DiamatTM Analyzer (ClinRep® kit, RECIPE Chemicals + Instruments GmbH, Munich, Germany).

**Mortality follow-up:** A mortality follow-up collects information on vital status from population registries at annual intervals. For this project, data were provided from the mortality follow-up conducted in 2009. For deceased persons, death certificates are requested from the local health authorities. Two internists independently validate the underlying cause of death and perform joint readings together with a third internist in cases of disagreement.

**Non-fatal myocardial infarction and stroke notification source and procedure:** In 2006 and 2008, postal questionnaires were sent to all study participants to assess incident cases of diseases including myocardial infarctions and strokes.

**Tromsø Study**

**Recruitment and data collection:** The Tromsø Study is a prospective study of inhabitants in the municipality of Tromsø, Norway. It is a repeated population-based health survey with examinations in 1974 (Tromsø 1), 1979-80 (Tromsø 2), 1986-87 (Tromsø 3), 1994-95 (Tromsø 4), 2001 (Tromsø 5), 2007-08 (Tromsø 6). Tromsø 4 is used as the baseline for this analysis. All inhabitants of Tromsø ≥ 25 years of age were invited to a screening and 27 159 subjects (77%) participated. The examinations included standardized measurements of height, weight, blood pressure, non-fasting serum lipids, and blood cell counts. A self-administered questionnaire handed in at the screening examination covered information about current and previous cigarette smoking, physical activity in leisure time, currently or previously treated hypertension, and a medical history of angina pectoris, diabetes mellitus, asthma, myocardial infarction, and stroke.

**Ethics committee(s) that approved the study:** Regional Committee of Medical and Health Research Ethics, North Norway.

**HbA_1c_ assay information:** The Cobas Mira instrument was used to quantify HbA_1c_ with an immunoturbidimetric method (Unimate 5 HbA_1c_, Hoffmann-La Roche, Basel, Swiss).

**Mortality follow-up:** Mortality was assessed until end of 2010 for this study via record linkage to Statistics Norway and the death certificates were retrieved.

**Non-fatal myocardial infarction and stroke notification source and procedure:** Hospitalized cases of incident myocardial infarction or stroke were identified by linking the Tromsø study participant list to the discharge diagnosis register at the University Hospital of North Norway, the only local hospital serving the Tromsø population. Discharge letters from hospitalizations in other hospitals were also collected.

All included cohort studies are conducted in accordance with the declaration of Helsinki as revised in 2008 and written informed consent was obtained from all study participants.

# Suppl. Table S1 – Source of information for selected baseline covariates

| Cohort |  | Weight and height | |  | History of diabetes | |  | History of hypertension | |  | History of MI or stroke | |  |
| --- | --- | --- | --- | --- | --- | --- | --- | --- | --- | --- | --- | --- | --- |
|  |  | Self- reported | Measured |  | Self- reported | Documented |  | Self- reported | Documented |  | Self- reported | Documented |  |
| ESTHER |  | X | X |  | X | X |  | X | X |  | X |  |  |
| SHIP |  |  | X |  | X |  |  | X |  |  | X |  |  |
| ELSA |  |  | X |  | X |  |  | X |  |  | X |  |  |
| Tromsø |  |  | X |  | X | X |  | X |  |  | X | X |  |
| KORA |  |  | X |  | X |  |  | X |  |  | X |  |  |
| NHANES |  | X | X |  | X |  |  | X |  |  | X |  |  |

X: Assessed.

Abbreviations: MI, myocardial infarction.

# Suppl. Table S2 – Proportion of missing values in analyzed samples from the six population-based cohorts

| **Characteristic** | **Proportion of missing values n (%)** | | | | | |
| --- | --- | --- | --- | --- | --- | --- |
|  | **ESTHER** | **SHIP** | **ELSA** | **Tromsø** | **KORA** | **NHANES** |
|  | **(n=7,982)** | **(n=1,777)** | **(n=5,262)** | **(n=6,045)** | **(n=1,850)** | **(n=5,778)** |
| Age | 0 (0) | 0 (0) | 0 (0) | 0 (0) | 0 (0) | 0 (0) |
| Sex | 0 (0) | 0 (0) | 0 (0) | 0 (0) | 0 (0) | 0 (0) |
| Race/ethnicity | N/A | N/A | N/A | N/A | N/A | 0 (0) |
| School education | 170 (2.1) | 11 (0.6) | 165 (3.1) | 39 (0.6) | 5 (0.3) | 50 (0.9) |
| BMI | 8 (0.1) | 2 (0.1) | 222 (4.2) | 12 (0.2) | 11 (0.6) | 28 (0.5) |
| Smoking | 210 (2.6) | 51 (2.8) | 45 (0.9) | 5 (0.1) | 34 (1.8) | 0 (0) |
| Alcohol consumption | **740 (9.3)** | **97 (5.5)** | 193 (3.7) | **1416 (23.4)** | 8 (0.4) | **463 (8.0)** |
| Vigorous physical activity | 20 (0.3) | 18 (1.0) | 2 (0.0) | 66 (1.1) | 8 (0.4) | 1 (0.0) |
| Total cholesterol | 25 (0.3) | 11 (0.6) | 38 (0.7) | 9 (0.1) | 1 (0.1) | 102 (1.8) |
| HDL cholesterol | **3025 (37.9)** | 11 (0.6) | 39 (0.7) | 16 (0.3) | 3 (0.2) | 123 (2.1) |
| Subclinical inflammation | 131 (1.6) | 71 (4.0) | 42 (0.8) | **849 (14.0)** | 19 (1.0) | 151 (2.6) |
| Serum creatinine | 25 (0.3) | 6 (0.3) | N/A | 13 (0.2) | 0 (0) | 199 (3.4) |
| Albuminuria | 37 (0.5) | **201 (11.3)** | N/A | **788 (13.0)** | N/A | **469 (8.1)** |
| Haemoglobin | N/A | 2 (0.1) | 14 (0.3) | 141 (2.3) | 0 (0) | 86 (1.5) |
| Hypertension | 1 (0) | 2 (0.1) | 0 (0) | 0 (0) | 7 (0.4) | 1 (0.0) |
| History of MI or stroke | 132 (1.7) | 12 (0.7) | 3 (0.1) | 0 (0) | 0 (0) | 0 (0) |
| GGT | N/A | N/A | N/A | N/A | N/A | **1496 (25.9)** |
| AST | N/A | N/A | N/A | N/A | N/A | 198 (3.4) |
| ALT | N/A | N/A | N/A | N/A | N/A | 198 (3.4) |
| HbA_1c_ | 0 (0) | 0 (0) | 0 (0) | 0 (0) | 0 (0) | 0 (0) |
| Ferritin | N/A | N/A | N/A | N/A | N/A | 81 (1.4) |
| Transferrin saturation | N/A | N/A | N/A | N/A | N/A | 78 (0.5) |
| Erythrocyte protoporphyrin | N/A | N/A | N/A | N/A | N/A | 29 (0.5) |

Bold printed: Missing values > 5%.

Abbreviations: ALT, Alanine transferase; AST, Aspartate transferase; BMI, body mass index; GGT, Gamma-glutamyltransferase; HbA_1c_, glycated haemoglobin; HDL, high-density lipoprotein; MI, myocardial infarction; N/A, not applicable; n, sample size.

# Suppl. Table S3 – Baseline characteristics of subjects without diabetes mellitus by intermediate and low HbA_1c_ levels, and multivariable adjusted odds ratios for associations of characteristics with intermediate HbA_1c_ levels. Pooled data from six cohort studies

| **Characteristic** | **Unit** | **Weighted mean or proportion [%] (95%CI)** | |  | **Pooled odds  ratio (95%CI)** |  | | | | |
| --- | --- | --- | --- | --- | --- | --- | --- | --- | --- | --- |
|  |  | **Intermediate HbA_1c_** | **Low HbA_1c_** |  |  |  | | | | |
|  |  | **(5.5- <6.0%) (37- <42 mmol/ mol)** | **(5.0- <5.5%) (31- <37 mmol/mol) (Reference group)** |  |  |  | | | | |
| Age | years | 63.5 (63.4-63.6) | 62.2 (62.1-62.4) |  | **1.28 (1.23; 1.33)  per 10 years** |  | | | | |
| Male sex | % | 44.0 (43.1-44.9) | 45.0 (44.1-46.0) |  | 0.97 (0.86; 1.09)* |  | | | | |
| Race/ethnicity ^1^ |  |  |  |  |  |  | | | | |
| Non-hispanic white | % | 56.7 | 70.0 |  | Ref. |  | | | | |
| Non-hispanic black | % | 19.0 | 11.8 |  | **2.27 (1.85; 2.77)** |  | | | | |
| Mexican-American | % | 20.0 | 15.1 |  | **1.86 (1.54; 2.25)** |  | | | | |
| Other | % | 4.2 | 3.0 |  | **1.99 (1.41; 2.80)** |  | | | | |
| School education |  |  |  |  |  |  | | | | |
| ≤ 9 years | % | 50.4 (49.4-51.4) | 45.9 (44.9-46.9) |  | Ref |  | | | | |
| 10 – 12 years | % | 37.1 (36.2-38.0) | 39.1 (38.1-40.0) |  | 1.00 (0.95; 1.04) |  | | | | |
| ≥ 13 years | % | 16.0 (15.3-16.7) | 19.0 (18.3-19.8) |  | 1.02 (0.92; 1.13) |  | | | | |
| BMI category |  |  |  |  |  |  | | | | |
| Underweight or low weight | % | 3.7 (3.3-4.1) | 3.9 (3.5-4.3) |  | 0.93 (0.83; 1.05) |  | | | | |
| Optimal weight | % | 28.2 (27.3-29.0) | 34.6 (33.7-35.5) |  | Ref |  | | | | |
| Overweight | % | 45.3 (44.4-46.1) | 43.6 (42.7-44.6) |  | 1.03 (0.98; 1.09) |  | | | | |
| Obesity | % | 23.9 (23.2-24.7) | 19.2 (18.5-20.0) |  | **1.23 (1.15; 1.32)** |  | | | | |
| Smoking |  |  |  |  |  |  | | | | |
| Never | % | 45.0 (44.1-45.9) | 45.8 (44.8-46.7) |  | Ref |  | | | | |
| Former | % | 33.3 (32.5-34.2) | 34.8 (33.9-35.7) |  | 1.05 (0.99; 1.12) |  | | | | |
| Current | % | 22.5 (21.7-23.3) | 20.0 (19.5-21.1) |  | **1.42 (1.31; 1.53)** |  | | | | |
| Relative alcohol consumption |  |  |  |  |  |  | | | | |
| Abstainer or low | % | 56.7 (55.8-57.6) | 50.9 (49.9-51.9) |  | Ref |  | | | | |
| Moderate | % | 34.3 (33.5-35.3) | 37.4 (36.4-38.3) |  | 1.02 (0.98; 1.07) |  | | | | |
| High | % | 9.0 (8.5-9.6) | 12.0 (11.4-12.7) |  | **0.85 (0.79; 0.91)** |  | | | | |
| Vigorous physical activity | % | 40.3 (39.4-41.2) | 42.3 (41.4-43.3) |  | 1.04 (0.98; 1.10) |  | | | | |
| Total cholesterol | mmol/L | 6.14 (6.11-6.16) | 6.03 (6.01-6.05) |  | **1.14 (1.11; 1.17)  per 1 mmol/L** |  | | | | |
| HDL cholesterol | mmol/L | 1.47 (1.46-1.47) | 1.52 (1.51-1.53) |  | **0.83 (0.77; 0.90)  per 1 mmol/L** |  | | | | |
| Subclinical inflammation | % | 35.0 (34.1-35.8) | 28.1 (27.2-29.0) |  | **1.14 (1.02; 1.29)*** |  | | | | |
| Serum creatinine | nmol/L | 77.7 (76.6-77.4) | 76.4 (76.0-76.8) |  | **1.02 (1.00; 1.03)  per 10 nmol/L** |  | | | | |
| Albuminuria | % | 15.6 (14.8-16.4) | 13.3 (12.5-14.1) |  | **1.11 (1.01; 1.22)** |  | | | | |
| Haemoglobin | g/dL | 14.04 (14.0-14.1) | 14.16 (14.14-14.19) |  | **0.88 (0.80; 0.97)*  per 1 g/dl** |  | | | | |
| Biomarkers of iron deficiency ^1^ |  |  |  |  |  |  | | | | |
| Ferritin | µg/L | 144 (138) | 152 (148) |  | 0.96 (0.92; 1.01)  per 100 µg/L |  | | | | |
| Transferrin saturation | % | 24.4 (9.7) | 26.0 (10.6) |  | **0.90 (0.84; 0.96)  per 10 %** |  | | | | |
| Erythrocyte protoporphyrin | µmol/L | 0.94 (0.41) | 0.93 (0.42) |  | 0.96 (0.89; 1.04)  per 0.5 µmol/L |  | | | | |
| **Suppl. Table S3 continues** | | | | | |  |  |  |  |  |
| **Characteristic** | **Unit** | **Weighted mean or proportion [%] (95%CI)** | |  | **Pooled odds  ratio (95%CI)** |  | | | | |
|  |  | **Intermediate HbA_1c_** | **Low HbA_1c_** |  |  |  | | | | |
|  |  | **(5.5- <6.0%) (37- <42 mmol/ mol)** | **(5.0- <5.5%) (31- <37 mmol/mol) (Reference group)** |  |  |  | | | | |
| Hypertension |  |  |  |  |  |  | | | | |
| No hypertension | % | 42.9 (42.0-43.8) | 47.0 (46.1-48.0) |  | Ref |  | | | | |
| Known hypertension or systolic  blood pressure ≥140-<160 mmHg | % | 43.6 (42.7-44.5) | 40.2 (39.3-41.1) |  | 1.00 (0.96; 1.05) |  | | | | |
| Systolic blood pressure ≥ 160  mmHg | % | 14.6 (13.9-15.2) | 14.0 (13.4-14.7) |  | 1.01 (0.96; 1.07) |  | | | | |
| History of MI or stroke | % | 8.9 (8.4-9.5) | 7.1 (6.6-7.6) |  | 1.17 (0.96; 1.42)* |  | | | | |
| Biomarkers of liver function ^1^ |  |  |  |  |  |  | | | | |
| GGT | U/L | 28.8 (29.5) | 29.9 (40.3) |  | 0.99 (0.97; 1.02)  per 10 U/L |  | | | | |
| AST | U/L | 21.1 (7.4) | 22.0 (11.9) |  | **0.83 (0.73; 0.93)  per 10 U/L** |  | | | | |
| ALT | U/L | 14.3 (8.5) | 14.6 (11.3) |  | **1.19 (1.05; 1.33)  per 10 U/L** |  | | | | |

Note: The table shows pooled means or proportions of baseline characteristics in the HbA_1c_ categories and additionally the results of a multivariable logistic regression model including all variables of the table as explanatory variables for intermediate HbA_1c_ (reference: low HbA_1c_).

Bold printed: Statistically significant difference (p<0.05). Abbreviations: ALT, Alanine transferase; AST, Aspartate transferase; BMI, body mass index; GGT, Gamma-glutamyltransferase; HbA_1c_, glycated haemoglobin; HDL, high-density lipoprotein; MI, myocardial infarction.

* Random effects model reported because of statistically significant heterogeneity.

^1^ Assessed only in NHANES.

# Suppl. Table S4 - Associations of HbA_1c_ levels with all-cause mortality in subjects without diabetes mellitus

| **Study** | **Very low HbA_1c_ (<5.0%) (<31 mmol/mol)** | | | | |  | **Low HbA_1c_ (5.0-< 5.5%; 31-<37 mmol/mol)** | | | |  | **Intermediate HbA_1c_ (5.5 - <6.0%) (37- <42 mmol/mol)** | | | | |  | **Increased HbA_1c_ (6.0 - <6.5%) (42- <48 mmol/mol)** | | | | |
| --- | --- | --- | --- | --- | --- | --- | --- | --- | --- | --- | --- | --- | --- | --- | --- | --- | --- | --- | --- | --- | --- | --- |
|  | **n_total_** | **n_cases_** | **IR ^1^** | **HR (95%CI)** | |  | **n_total_** | **n_cases_** | **IR ^1^** | **HR** |  | **n_total_** | **n_cases_** | **IR ^1^** | **HR (95%CI)** | |  | **n_total_** | **n_cases_** | **IR ^1^** | **HR (95%CI)** | |
|  |  |  |  | **Simple  model^2^** | **Full  model^3^** |  |  |  |  |  |  |  |  |  | **Simple  model^2^** | **Full  model^3^** |  |  |  |  | **Simple  model^2^** | **Full  model^3^** |
| ESTHER | 476 | 54 | 10.0 | 1.12  (0.83; 1.48) | 1.20  (0.90; 1.61) |  | 2746 | 312 | 10.1 | Ref |  | 3726 | 504 | 12.2 | 1.14  (0.99; 1.31) | 1.07  (0.93; 1.24) |  | 1033 | 199 | 18.0 | 1.58  (1.32; 1.88) | 1.29  (1.08; 1.55) |
| SHIP | 336 | 40 | 12.6 | 0.88 (0.60; 1.27) | 0.95 (0.65; 1.38) |  | 546 | 94 | 18.4 | Ref |  | 583 | 93 | 16.6 | 0.77 (0.58; 1.03) | 0.74 (0.55; 0.98) |  | 312 | 71 | 24.5 | 1.06 (0.78; 1.44) | 0.88 (0.64; 1.21) |
| ELSA | 344 | 37 | 15.3 | 1.27 (0.88; 1.78) | 1.20 (0.84; 1.70) |  | 2339 | 239 | 14.5 | Ref |  | 2199 | 302 | 19.9 | 1.13 (0.95; 1.34) | 0.99 (0.83; 1.18) |  | 371 | 67 | 27.1 | 1.30 (0.98; 1.70) | 0.95 (0.71; 1.26) |
| KORA | 63 | 6 | 11.7 | 1.04 (0.42; 2.24) | 0.98 (0.41; 2.31) |  | 603 | 50 | 9.9 | Ref |  | 937 | 91 | 11.2 | 1.01 (0.72; 1.44) | 0.99 (0.70; 1.40) |  | 247 | 33 | 15.5 | 1.38 (0.88; 2.13) | 1.33 (0.85; 2.08) |
| NHANES | 677 | 325 | 41.1 | 1.20 (1.06; 1.36) | 1.15 (1.01; 1.31) |  | 1992 | 963 | 41.1 | Ref |  | 2272 | 1135 | 44.3 | 1.06 (0.97; 1.15) | 1.04 (0.95; 1.13) |  | 834 | 450 | 50.8 | 1.19 (1.06; 1.33) | 1.12 (1.00; 1.26) |
| Tromsø | 626 | 129 | 14.2 | 0.85 (0.70; 1.02) | 0.82 (0.68; 0.99) |  | 2719 | 727 | 19.0 | Ref |  | 2323 | 705 | 21.8 | 1.01 (0.91; 1.13) | 0.97 (0.87; 1.08) |  | 377 | 143 | 29.2 | 1.33 (1.11; 1.59) | 1.25 (1.04; 1.50) |
| **Meta-analysis fixed effects** | **2522** | **591** | **20.5** | **1.08 (0.98; 1.18)** | **1.06 (0.96; 1.16)** |  | **10945** | **2385** | **19.3** | **Ref** |  | **12040** | **2830** | **21.7** | **1.05 (0.99; 1.11)** | **1.00 (0.95; 1.06)** |  | **3174** | **963** | **29.5** | **1.28 (1.19; 1.38)** | **1.15 (1.06; 1.24)** |
| **Meta-analysis  random effects** | **2522** | **591** | **20.5** | **1.05 (0.89; 1.24)** | **1.04 (0.89; 1.22)** |  | **10945** | **2385** | **19.3** | **Ref** |  | **12040** | **2830** | **21.7** | **1.05 (0.98; 1.12)** | **1.00 (0.94; 1.07)** |  | **3174** | **963** | **29.5** | **1.30 (1.16; 1.46)** | **1.14 (1.03; 1.27)** |
| **Heterogeneity (I²; p)** |  |  |  | **55.7 %;  0.046** | **51.6 %; 0.066** |  |  |  |  |  |  |  |  |  | **26.0 %; 0.239** | **18.0 %; 0.297** |  |  |  |  | **42.1 %; 0.125** | **32.1 %; 0.195** |

Abbreviations: CI, confidence interval; HbA_1c_, glycated haemoglobin; HR, hazard ratio; IR, incidence rate; n, sample size; Ref, reference.

^1^ IR= Incidence rate per 1,000 person-years. The IR for the meta-analyses is the weighted mean of the IRs of the individual studies (weighted by sample size).

^2^ Adjusted for age and sex.

^3^ Adjusted for age, sex, race/ethnicity, BMI, education, smoking, physical activity, alcohol consumption, total cholesterol, HDL cholesterol, CRP, haemoglobin, serum creatinine, albuminuria, hypertension and history of CVD.

Suppl. Table S5 - Associations of HbA_1c_ levels with cardiovascular mortality in subjects without diabetes mellitus

| **Study** | **Very low HbA_1c_ (<5.0%) (<31 mmol/mol)** | | | | |  | **Low HbA_1c_ (5.0-< 5.5%; 31-<37 mmol/mol)** | | | |  | **Intermediate HbA_1c_ (5.5 - <6.0%) (37- <42 mmol/mol)** | | | | |  | **Increased HbA_1c_ (6.0 - <6.5%) (42- <48 mmol/mol)** | | | | |
| --- | --- | --- | --- | --- | --- | --- | --- | --- | --- | --- | --- | --- | --- | --- | --- | --- | --- | --- | --- | --- | --- | --- |
|  | **n_total_** | **n_cases_** | **IR ^1^** | **HR (95%CI)** | |  | **n_total_** | **n_cases_** | **IR ^1^** | **HR** |  | **n_total_** | **n_cases_** | **IR ^1^** | **HR (95%CI)** | |  | **n_total_** | **n_cases_** | **IR ^1^** | **HR (95%CI)** | |
|  |  |  |  | **Simple  model^2^** | **Full  model^3^** |  |  |  |  |  |  |  |  |  | **Simple  model^2^** | **Full  model^3^** |  |  |  |  | **Simple  model^2^** | **Full  model^3^** |
| ESTHER | 474 | 18 | 3.4 | 1.80  (1.04; 2.95) | 2.08  (1.23; 3.51) |  | 2735 | 69 | 2.2 | Ref |  | 3708 | 125 | 3.0 | 1.21  (0.90; 1.63) | 1.17  (0.87; 1.57) |  | 1026 | 51 | 4.6 | 1.67  (1.16; 2.40) | 1.32  (0.91; 1.92) |
| SHIP | 332 | 5 | 1.6 | 0.41 (0.14; 0.97) | 0.45 (0.17; 1.18) |  | 542 | 27 | 5.3 | Ref |  | 578 | 32 | 5.8 | 0.90 (0.54; 1.51) | 0.83 (0.49; 1.41) |  | 309 | 26 | 9.1 | 1.36 (0.79; 2.34) | 1.05 (0.60; 1.85) |
| ELSA | 338 | 8 | 3.4 | 1.15 (0.51; 2.27) | 0.98 (0.47; 2.08) |  | 2297 | 63 | 3.9 | Ref |  | 2123 | 85 | 5.8 | 1.14 (0.82; 1.59) | 0.97 (0.69; 1.36) |  | 355 | 18 | 7.6 | 1.25 (0.72; 2.07) | 0.69 (0.39; 1.22) |
| KORA | 63 | 1 | 2.0 | 0.56 (0.07; 2.77) | 0.58 (0.07; 4.49) |  | 601 | 15 | 3.0 | Ref |  | 933 | 38 | 4.7 | 1.35 (0.76; 2.54) | 1.32 (0.72; 2.43) |  | 246 | 15 | 7.0 | 2.01 (0.97; 4.16) | 1.89 (0.91; 3.92) |
| NHANES | 670 | 139 | 17.7 | 1.22 (1.01; 1.48) | 1.17 (0.97; 1.42) |  | 1968 | 428 | 18.4 | Ref |  | 2255 | 573 | 22.5 | 1.18 (1.05; 1.34) | 1.15 (1.01; 1.30) |  | 828 | 205 | 23.3 | 1.20 (1.01; 1.41) | 1.11 (0.94; 1.32) |
| Tromsø | 622 | 59 | 6.5 | 0.97 (0.72; 1.27) | 0.90 (0.68; 1.19) |  | 2695 | 302 | 7.9 | Ref |  | 2295 | 275 | 8.6 | 0.93 (0.79; 1.09) | 0.87 (0.74; 1.02) |  | 375 | 71 | 14.5 | 1.52 (1.16; 1.95) | 1.29 (0.99; 1.68) |
| **Meta-analysis fixed effects** | **2499** | **230** | **7.7** | **1.14 (0.99; 1.32)** | **1.11 (0.95; 1.28)** |  | **10838** | **904** | **7.1** | **Ref** |  | **11892** | **1128** | **8.5** | **1.10 (1.00; 1.20)** | **1.04 (0.95; 1.14)** |  | **3139** | **386** | **11.7** | **1.34 (1.19; 1.51)** | **1.16 (1.02; 1.32)** |
| **Meta-analysis  random effects** | **2499** | **230** | **7.7** | **1.11 (0.84; 1.45)** | **1.08 (0.79; 1.47)** |  | **10838** | **904** | **7.1** | **Ref** |  | **11892** | **1128** | **8.5** | **1.09 (0.97; 1.23)** | **1.03 (0.89; 1.19)** |  | **3139** | **386** | **11.7** | **1.35 (1.19; 1.55)** | **1.17 (1.00; 1.37)** |
| **Heterogeneity (I²; p)** |  |  |  | **48.0 %; 0.087** | **57.6 %; 0.038** |  |  |  |  |  |  |  |  |  | **28.4 %; 0.222** | **43.3 %; 0.117** |  |  |  |  | **6.9 %; 0.373** | **20.8 %; 0.277** |

Abbreviations: CI, confidence interval; HbA_1c_, glycated haemoglobin; HR, hazard ratio; IR, incidence rate; n, sample size; Ref, reference.

^1^ IR= Incidence rate per 1,000 person-years. The IR for the meta-analyses is the weighted mean of the IRs of the individual studies (weighted by sample size).

^2^ Adjusted for age and sex.

^3^ Adjusted for age, sex, race/ethnicity, BMI, education, smoking, physical activity, alcohol consumption, total cholesterol, HDL cholesterol, CRP, haemoglobin, serum creatinine, albuminuria, hypertension and history of CVD.

Suppl. Table S6 - Associations of HbA_1c_ levels with cardiovascular events in subjects without diabetes mellitus

| **Study** | **Very low HbA_1c_ (<5.0%) (<31 mmol/mol)** | | | | |  | **Low HbA_1c_ (5.0-<5.5%) (31- <37 mmol/mol)** | | | |  | **Intermediate HbA_1c_ (5.5 - <6.0%) (37- <42 mmol/mol)** | | | | |  | **Increased HbA_1c_ (6.0 - <6.5%) (42- <48 mmol/mol)** | | | | |
| --- | --- | --- | --- | --- | --- | --- | --- | --- | --- | --- | --- | --- | --- | --- | --- | --- | --- | --- | --- | --- | --- | --- |
|  | **n_total_** | **n_cases_** | **IR ^1^** | **HR (95%CI)** | |  | **n_total_** | **n_cases_** | **IR ^1^** | **HR** |  | **n_total_** | **n_cases_** | **IR ^1^** | **HR (95%CI)** | |  | **n_total_** | **n_cases_** | **IR ^1^** | **HR (95%CI)** | |
|  |  |  |  | **Simple  model^2^** | **Full  model^3^** |  |  |  |  |  |  |  |  |  | **Simple  model^2^** | **Full  model^3^** |  |  |  |  | **Simple  model^2^** | **Full  model^3^** |
| ESTHER | 439 | 29 | 9.0 | 1.00  (0.66; 1.46) | 1.08  (0.73; 1.60) |  | 2515 | 179 | 9.9 | Ref |  | 3376 | 292 | 12.4 | 1.20  (1.00; 1.45) | 1.12  (0.93; 1.35) |  | 903 | 95 | 15.5 | 1.43  (1.11; 1.84) | 1.18  (0.92; 1.53) |
| SHIP | 312 | 3 | 1.0 | 0.33 (0.10; 0.97) | 0.39 (0.11; 1.34) |  | 504 | 19 | 4.0 | Ref |  | 539 | 24 | 4.6 | 0.96 (0.53; 1.78) | 0.92 (0.50; 1.71) |  | 282 | 19 | 7.2 | 1.48 (0.78; 2.82) | 1.27 (0.65; 2.45) |
| ELSA | 303 | 13 | 7.9 | 0.83 (0.45; 1.42) | 0.83 (0.47; 1.48) |  | 2051 | 128 | 11.7 | Ref |  | 1826 | 136 | 14.4 | 1.12 (0.88; 1.43) | 1.01 (0.79; 1.30) |  | 282 | 38 | 26.4 | 1.86 (1.28; 2.65) | 1.45 (0.99; 2.12) |
| KORA | 53 | 2 | 4.8 | 0.54 (0.13; 1.77) | 0.56 (0.13; 2.35) |  | 503 | 34 | 8.4 | Ref |  | 795 | 72 | 10.9 | 1.18 (0.79; 1.80) | 1.14 (0.75; 1.73) |  | 205 | 24 | 14.2 | 1.51 (0.88; 2.54) | 1.39 (0.82; 2.36) |
| NHANES | N/A | N/A | N/A | N/A | N/A |  | N/A | N/A | N/A | Ref |  | N/A | N/A | N/A | N/A | N/A |  | N/A | N/A | N/A | N/A | N/A |
| Tromsø | 578 | 112 | 14.6 | 0.90 (0.73; 1.10) | 0.90 (0.73; 1.10) |  | 2544 | 548 | 18.1 | Ref |  | 2141 | 581 | 21.9 | 1.10 (0.98; 1.24) | 1.00 (0.89; 1.13) |  | 339 | 109 | 27.6 | 1.34 (1.09; 1.64) | 1.10 (0.89; 1.35) |
| **Meta-analysis fixed effects** | **1685** | **159** | **9.1** | **0.88 (0.75; 1.05)** | **0.90 (0.76; 1.07)** |  | **8117** | **908** | **12.5** | **Ref** |  | **8677** | **1105** | **14.5** | **1.13 (1.03; 1.23)** | **1.03 (0.95; 1.13)** |  | **2011** | **285** | **17.8** | **1.45 (1.27; 1.67)** | **1.19 (1.04; 1.37)** |
| **Meta-analysis  random effects** | **1685** | **159** | **9.1** | **0.88 (0.75; 1.05)** | **0.90 (0.76; 1.07)** |  | **8117** | **908** | **12.5** | **Ref** |  | **8677** | **1105** | **14.5** | **1.13 (1.03; 1.23)** | **1.03 (0.95; 1.13)** |  | **2011** | **285** | **17.8** | **1.45 (1.27; 1.67)** | **1.19 (1.04; 1.37)** |
| **Heterogeneity (I²; p)** |  |  |  | **0 %; 0.416** | **0 %; 0.546** |  |  |  |  |  |  |  |  |  | **0 %; 0.923** | **0 %; 0.860** |  |  |  |  | **0 %; 0.664** | **0 %; 0.747** |

Abbreviations: CI, confidence interval; HbA_1c_, glycated haemoglobin; HR, hazard ratio; IR, incidence rate; N/A, not applicable; n, sample size; Ref, reference.

^1^ IR= Incidence rate per 1,000 person-years. The IR for the meta-analyses is the weighted mean of the IRs of the individual studies (weighted by sample size).

^2^ Adjusted for age and sex.

^3^ Adjusted for age, sex, BMI, education, smoking, physical activity, alcohol consumption, total cholesterol, HDL cholesterol, CRP, haemoglobin, serum creatinine, albuminuria, hypertension and history of CVD.

# Suppl. Table S7 – Sex stratified analyses of the associations of HbA_1c_ levels with mortality and cardiovascular outcomes in subjects without diabetes mellitus

| **Outcome/** | **Very low HbA_1c_ (<5.0%) (<31 mmol/mol)** | | | |  | **Low HbA_1c_ (5.0 - <5.5%) (31- <37 mmol/mol)** | | | |  | | **Intermediate HbA_1c_ (5.5 - <6.0%) (37- <42 mmol/mol)** | | | |  | **Increased HbA_1c_ (6.0 - <6.5%) (42- <48 mmol/mol)** | | | |
| --- | --- | --- | --- | --- | --- | --- | --- | --- | --- | --- | --- | --- | --- | --- | --- | --- | --- | --- | --- | --- |
| **Stratum** | **n_total_** | **n_cases_** | **IR ^1^** | **HR (95%CI) ^2^** |  | **n_total_** | **n_cases_** | **IR ^1^** | **HR** | |  | **n_total_** | **n_cases_** | **IR ^1^** | **HR (95%CI) ^2^** |  | **n_total_** | **n_cases_** | **IR ^1^** | **HR (95%CI) ^2^** |
| **All-cause mortality** | | | | |  |  |  |  |  | |  |  |  |  |  |  |  |  |  |  |
| Women | 1255 | 222 | 15.6 | 1.04 (0.90; 1.21) |  | 5732 | 1039 | 16.1 | Ref | |  | 6260 | 1302 | 19.0 | 1.04 (0.96; 1.14) |  | 1552 | 385 | 23.1 | 1.08 (0.95; 1.22) |
| Men | 1204 | 363 | 28.4 | 1.07 (0.90; 1.23) |  | 4610 | 1296 | 25.5 | Ref | |  | 4843 | 1437 | 28.3 | 0.95 (0.88; 1.03) |  | 1375 | 545 | 40.5 | **1.18 (1.06; 1.32)** |
| **Cardiovascular mortality** | | | | |  |  |  |  |  | |  |  |  |  |  |  |  |  |  |  |
| Women | 1247 | 90 | 6.3 | 1.14 (0.90; 1.44) |  | 5687 | 392 | 6.1 | Ref | |  | 6189 | 540 | 8.0 | 1.12 (0.98; 1.28) |  | 1537 | 167 | 9.9 | 1.12 (0.93; 1.36) |
| Men | 1189 | 139 | 10.8 | 1.11 (0.91; 1.34) |  | 4550 | 497 | 10.1 | Ref | |  | 4774 | 550 | 10.9 | 0.95 (0.84; 1.08) |  | 1356 | 204 | 15.3 | 1.13 (0.95; 1.34) |
| **Cardiovascular events** | | | | | | |  |  |  | |  |  |  |  |  |  |  |  |  |  |
| Women | 731 | 62 | 8.0 | 0.92 (0.70; 1.20) |  | 4124 | 400 | 10.0 | Ref | |  | 4390 | 492 | 12.6 | 1.07 (0.93; 1.23) |  | 881 | 113 | 14.9 | 1.16 (0.93; 1.44) |
| Men | 589 | 92 | 15.6 | 0.91 (0.73; 1.14) |  | 2986 | 480 | 19.8 | Ref | |  | 2953 | 517 | 20.4 | 1.00 (0.88; 1.14) |  | 643 | 129 | 27.2 | 1.17 (0.95; 1.62) |

Abbreviations: CI, confidence interval; HbA_1c_, glycated haemoglobin; HR, hazard ratio; IR, incidence rate; n, sample size.

^1^ IR= Incidence rate per 1,000 person-years. The IR is the weighted mean of the IRs of the individual studies (weighted by sample size).

# ^2^ Adjusted for age, sex, race/ethnicity, BMI, education, smoking, physical activity, alcohol consumption, total cholesterol, HDL cholesterol, CRP, haemoglobin, serum creatinine, albuminuria, hypertension and history of CVD.

# Suppl. Table S8 – Associations of potential confounders with all-cause mortality and cardiovascular mortality in subjects without diabetes mellitus in the NHANES in a model adjusted for age and sex

| **Characteristic** | **All-cause mortality** |  | **CV mortality** |
| --- | --- | --- | --- |
|  | **HR (95%CI)** |  | **HR (95%CI)** |
| Age | **2.70 (2.60; 2.81)**  per 10 years |  | **3.29 (3.10; 3.49)**  per 10 years |
| Male sex | **1.57 (1.46; 1.70)** |  | **1.49 (1.34; 1.66)** |
| Race/ethnicity |  |  |  |
| Non-Hispanic white | Ref. |  | Ref. |
| Non-Hispanic black | **1.20 (1.09; 1.33)** |  | **1.19 (1.03; 1.38)** |
| Mexican-American | **0.87 (0.78; 0.97)** |  | 0.87 (0.73; 1.02) |
| Other | **0.78 (0.61; 0.98)** |  | 0.84 (0.58; 1.17) |
| School education |  |  |  |
| ≤ 9 years | Ref |  | Ref |
| 10 – 12 years | 0.97 (0.89; 1.05) |  | 1.02 (0.90; 1.15) |
| ≥ 13 years | **0.80 (0.72; 0.88)** |  | **0.84 (0.73; 0.97)** |
| BMI category |  |  |  |
| Underweight or low weight | **1.79 (1.56; 2.05)** |  | **1.58 (1.28; 1.93)** |
| Optimal weight | Ref |  | Ref |
| Overweight | **0.90 (0.82; 0.98)** |  | 0.96 (0.85; 1.09) |
| Obesity | 0.94 (0.85; 1.05) |  | 1.07 (0.92; 1.26) |
| Smoking |  |  |  |
| Never | Ref |  | Ref |
| Former | **1.23 (1.13; 1.35)** |  | 1.13 (0.99; 1.28) |
| Current | **2.08 (1.88; 2.31)** |  | **1.74 (1.48; 2.03)** |
| Relative alcohol consumption |  |  |  |
| Abstainer or low | Ref |  | Ref |
| Moderate | 0.96 (0.90; 1.03) |  | **0.86 (0.74; 0.997)** |
| High | 1.08 (0.98; 1.20) |  | 1.18 (0.95; 1.45) |
| Vigorous physical activity | **0.74 (0.68; 0.80)** |  | **0.74 (0.65; 0.83)** |
| Total cholesterol | **0.96 (0.92; 0.99)** per 1 mmol/L |  | 1.03 (0.98; 1.08) per 1 mmol/L |
| HDL cholesterol | 1.05 (0.95; 1.15)  per 1 mmol/L |  | 0.98 (0.85; 1.13)  per 1 mmol/L |
| Subclinical inflammation | **1.29 (1.19; 1.39)** |  | **1.27 (1.13; 1.42)** |
| Serum creatinine | **1.05 (1.04; 1.07)** per 10 nmol/L |  | **1.07 (1.05; 1.08)** per 10 nmol/L |
| Albuminuria | **1.62 (1.49; 1.77)** |  | **1.66 (1.47; 1.88)** |
| Haemoglobin | **0.91 (0.88; 0.94)** per 1 g/dl |  | **0.92 (0.88; 0.96)** per 1 g/dl |
| Biomarkers of iron deficiency |  |  |  |
| Ferritin | 1.02 (0.998; 1.04)  per 100 µg/L |  | 1.02 (0.99; 1.05)  per 100 µg/L |
| Transferrin saturation | **0.95 (0.92; 0.99)**  per 10 % |  | **0.92 (0.87; 0.97)** per 10 % |
| Erythrocyte protoporphyrin | **1.03 (1.01; 1.06)**  per 0.5 µmol/L |  | 1.02 (0.99; 1.06)  per 0.5 µmol/L |
| **Suppl. Table S8 continues** |  |  |  |
| **Characteristic** | **All-cause mortality** |  | **CV mortality** |
|  | **HR (95%CI)** |  | **HR (95%CI)** |
| Hypertension |  |  |  |
| No hypertension | Ref |  | Ref |
| Known hypertension or  systolic blood pressure  ≥ 140- <160 mmHg | **1.13 (1.03; 1.23)** |  | **1.23 (1.08; 1.40)** |
| Systolic blood pressure  ≥ 160 mmHg | **1.41 (1.27; 1.58)** |  | **1.71 (1.46; 2.00)** |
| History of MI or stroke | **1.57 (1.43; 1.73)** |  | **2.04 (1.80; 2.32)** |
| Biomarkers of liver function |  |  |  |
| GGT | **1.02 (1.01; 1.03)** per 10 U/L |  | **1.02 (1.01; 1.03)**  per 10 U/L |
| AST | **1.05 (1.02; 1.08)**  per 10 U/L |  | 0.96 (0.89; 1.03)  per 10 U/L |
| ALT | **0.94 (0.89; 0.99)**  per 10 U/L |  | **0.82 (0.75; 0.90)**  per 10 U/L |

Bold printed: Statistically significant difference (p<0.05).

Abbreviations: ALT, Alanine transferase; AST, Aspartate transferase; BMI, body mass index; GGT, Gamma-glutamyltransferase; CI, confidence interval; HbA_1c_, glycated hemoglobin; HDL, high-density lipoprotein; HR, hazard ratio; MI, myocardial infarction.

# Suppl. Table S9 – Association of very low HbA_1c_ levels (<5%) with all-cause mortality in subjects without diabetes mellitus from NHANES stratified by 5-year age-groups (comparison group: Low HbA_1c_ (5.0- <5.5%))

| **Age (years)** | **HR (95%CI) ^a^** | | |
| --- | --- | --- | --- |
|  |  | **All-cause mortality** | **CV mortality** |
| **50 - 55** |  | 0.92 (0.55; 1.54) | 1.51 (0.60; 3.81) |
| **>55 - 60** |  | 1.24 (0.78; 1.98) | 0.52 (0.17; 1.60) |
| **>60 - 65** |  | 1.02 (0.71; 1.49) | 0.97 (0.54; 1.73) |
| **>65 - 70** |  | 1.07 (0.74; 1.55) | 1.17 (0.65; 2.11) |
| **>70 - 75** |  | 0.91 (0.65; 1.26) | 1.01 (0.61; 1.66) |
| **>75 - 80** |  | 0.99 (0.70; 1.38) | 1.00 (0.62; 1.62) |
| **>80 - 85** |  | 1.28 (0.94; 1.75) | 1.55 (1.03; 2.33) |
| **>85** |  | 1.12 (0.74; 1.70) | 0.99 (0.56; 1.76) |
| **Pooled strata ^b^** |  | 1.06 (0.93; 1.22) | 1.12 (0.92; 1.37) |

Abbreviations: CI, confidence interval; HbA_1c_, glycated haemoglobin; HR, hazard ratio; IR, incidence rate; n, sample size; Ref, reference.

^a^ Adjusted for sex, race/ethnicity, BMI, alcohol consumption, ferritin, erythrocyte protoporphyrin, gamma-glutamyltransferase and alanine transferase.

^b^ Fixed effects model to summarize the strata.


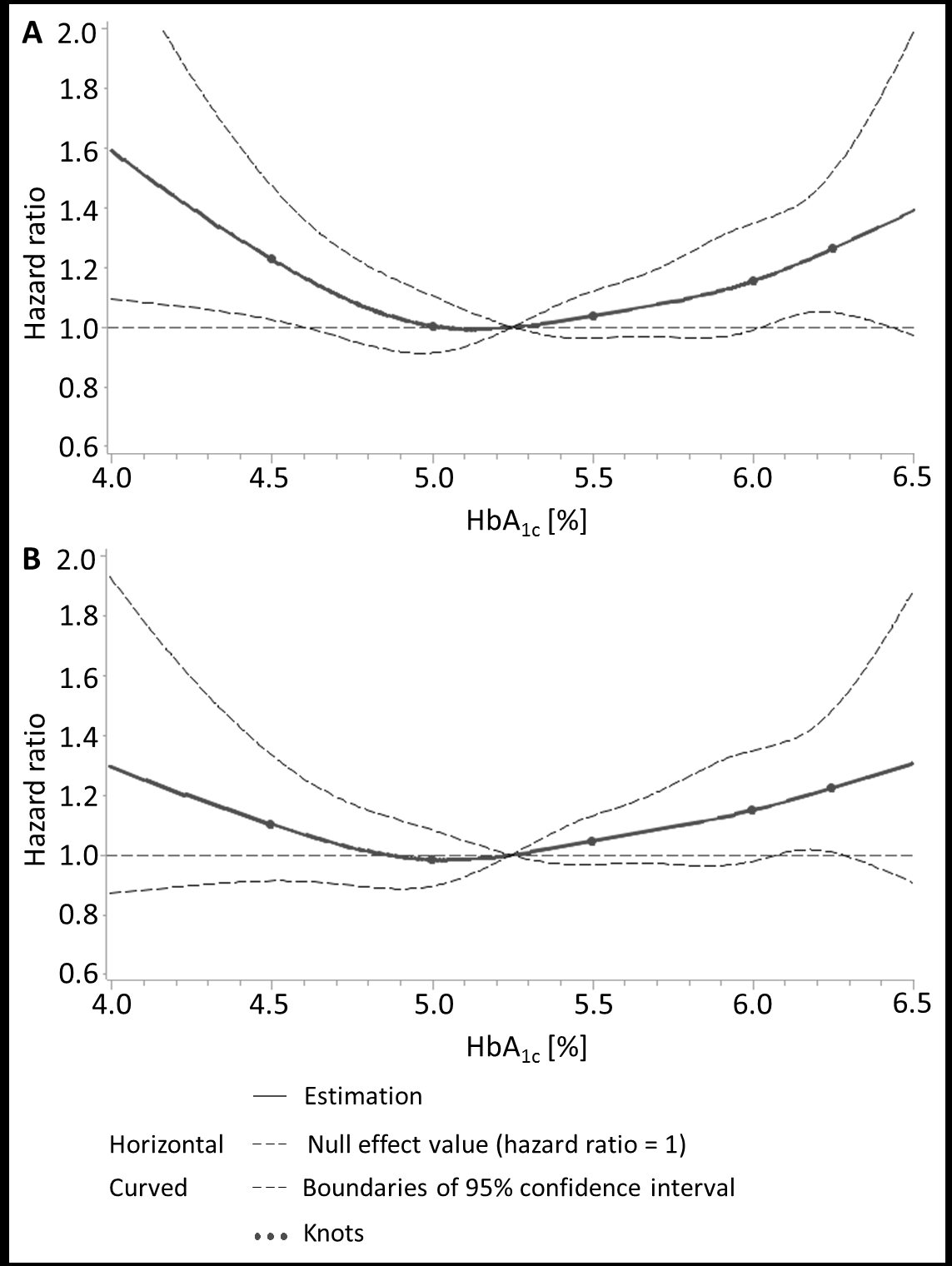


**Suppl. Figure 1** - Dose-response relationship of HbA_1c_ levels with cardiovascular mortality in subjects without diabetes mellitus in the NHANES with (A) adjustment for age and sex and (B) adjustment for all potential confounders (full model)

**Further funding sources of participating cohorts**

The **ESTHER** study was funded by the Baden-Württemberg state Ministry of Science, Research and Arts (Stuttgart, Germany), the Federal Ministry of Education and Research (Berlin, Germany) and the Federal Ministry of Family Affairs, Senior Citizens, Women and Youth (Berlin, Germany). Measurements of 25(OH)D in men were conducted in the context of the German Cancer Aid project number 108250 and 108426.

The **Tromsø Study** was performed by the University of Tromsø in cooperation with the National Health Screening Service. The Study was funded by the Norwegian Research Council.

The **KORA** cohort study was financed by the Helmholtz Zentrum München, German Research Center for Environmental Health (HMGU), which is funded by the German Federal Ministry of Education and Research (BMBF), and by the State of Bavaria. Additional funds were provided by the Federal Ministry of Health (BMG), the University of Ulm and the Ministry of Innovation, Science, Research, and Technology of the state North Rhine Westphalia (MIWF NRW) and a grant from the BMBF to the German Center for Diabetes Research (DZD e.V.). For this analysis, data of the KORA study were provided by the MORGAM Data Centre at THL, Helsinki, Finland. CHANCES funding was used for data coding and transfer.

The **SHIP** study is funded by following institutions: Federal Ministry of Education and Research (grants 01ZZ9603, 01ZZ0103, 01ZZ0403, 01ZZ0701**,** 03ZIK012), Ministry of Cultural Affairs as well as the Social Ministry of the Federal State of Mecklenburg-West Pomerania, Federal Ministry of Nutrition, Agriculture and Consumer’s Safety (07HS003), German Research Foundation (projects Gr 1912/5-1, Ko 799/5-1, Vo 955/5-1, Vo 955/6-1, Vo 955/10-1), Competence Network Heart Failure (01GI0205), Competence Network Diabetes (01GI0855), German Asthma and COPD Network (COSYCONET; BMBF 01GI0883), Genopathomik (BMBF FZK 03138010), Alfried Krupp von Bohlen und Halbach Foundation, Alexander v. Humboldt Foundation, Leibniz Society, Siemens AG, Health Care Sector (Erlangen, Germany), Pfizer Pharma GmbH (SBU Endocrinology and Ophthalmology; Berlin Germany), Novo Nordisk (Mainz, Germany), Data Input GmbH (Darmstadt, Germany), GABA International AG (Therwil, Switzerland), Imedos Systems (Jena, Germany) and Heinen and Löwenstein (Bad Ems, Germany).

The **ELSA** study is funded by a consortium of UK Government departments involved in areas related to the ageing process and the National Institute on Aging in the US.

**NHANES** is a US-governmental funded program of the National Center for Health Statistics (NCHS), USA. NCHS is part of the Centers for Disease Control and Prevention (CDC) and has the responsibility for producing vital and health statistics for the Nation.
